# Challenges of Current Anticancer Treatment Approaches with Focus on Liposomal Drug Delivery Systems

**DOI:** 10.3390/ph14090835

**Published:** 2021-08-24

**Authors:** Vijay Gyanani, Jeffrey C. Haley, Roshan Goswami

**Affiliations:** 1 Long Acting Drug Delivery, Celanese Corporation, Irving, TX 75039, USA; jeff.haley@celanese.com; 2 Formulation, R&D, mAbxience, 24009 Leon, Spain; roshan.goswami30@gmail.com

**Keywords:** chemotherapy, radiotherapy, active targeting, passive targeting, tumor, immunoconjugate, traditional liposome, stealth liposome, triggered release, limitations of liposomes

## Abstract

According to a 2020 World Health Organization report (Globocan 2020), cancer was a leading cause of death worldwide, accounting for nearly 10 million deaths in 2020. The aim of anticancer therapy is to specifically inhibit the growth of cancer cells while sparing normal dividing cells. Conventional chemotherapy, radiotherapy and surgical treatments have often been plagued by the frequency and severity of side effects as well as severe patient discomfort. Cancer targeting by drug delivery systems, owing to their selective targeting, efficacy, biocompatibility and high drug payload, provides an attractive alternative treatment; however, there are technical, therapeutic, manufacturing and clinical barriers that limit their use. This article provides a brief review of the challenges of conventional anticancer therapies and anticancer drug targeting with a special focus on liposomal drug delivery systems.

## 1. Introduction

### Cancer Statistics: Need for Better Therapeutics

Cancer is a group of diseases characterized by uncontrolled growth of abnormal cells that have the latent potential to penetrate other tissues. It is the leading cause of death worldwide, amounting to nearly 7.6 million deaths globally i.e., nearly 13% of total deaths in 2008 [1] and more recently 10 million deaths in 2020 [2]. As per current estimates, the number of cancer cases may reach an unprecedented 22.2 million in 2030 [1]. Statistics in the United States are no different where cancer is the second most prevalent cause of death, next to only heart related diseases [3]. As per the American Cancer Society, about 608,570 Americans are expected to die of cancer in 2021, which accounts for approximately 1670 deaths per day and nearly a quarter of total deaths in the US [3]. These data highlight the significance of anticancer research and the necessity to discover innovative ways to treat cancer.

The main goal of anticancer therapy is to specifically inhibit the malignant activity of cancer cells, while leaving healthy cells unaffected. Conventional anticancer treatments, including chemotherapy, radiotherapy and surgery, are challenged by drug resistance, severity and side effects. Some of the challenges and limitations of these therapies are discussed.

## 2. Limitations and Challenges Associated with Traditional Anticancer Therapies

### 2.1. Cancer Surgery

Cancer surgery is perceived to be an effective tool for eliminating early-stage cancer i.e., at the tumor level. However, it is worth acknowledging that not all early-stage cancerous tissues can be surgically removed. The limitation of surgery lies in how deep seated a tumor tissue is as well as its size. If the tumor size is perilously big, it can seriously impair the regular functioning of a surrounding tissue or organ. A relevant example, post brain surgery, is negative impact on normal functioning of brain i.e., thinking, speaking, etc. In this situation, surgery may not be a first preference for treatment [4]. Another pertinent example is breast cancer where accurate determination of tumor size and position remains a challenge and, therefore, limits the success of a surgical procedure [4].

Other notable examples where surgery impacts normal functioning include permanent impairment of fertility that may be caused by prostrate, ovarian and uterine surgery [4]. Similarly, impact on vocal cords caused by lung surgery performed especially in the upper trachea and shortness of breath developed after lower lung procedures are other known examples [4].

Furthermore, while there are other glaring instances, such as Laryngectomy which eliminates the natural ability to speak, procedures such as a Glossectomy do not eliminate natural speaking but lead to slurred speech with difficulty in swallowing [4].

Irrespective of the complications associated with cancer surgery at various sites, surgery inherently carry risks such as infections, bleeding and pain associated with local nerve injury.

### 2.2. Chemotherapy

Chemotherapy is a treatment regime where a combination of drugs is administered to the body. Notably, chemotherapy remains one of only a few treatment choices for advanced-stage cancer (metastasized cancer); however, a serious deficiency of chemotherapy is the lack of its target selectivity. As the cancer cells arise from normal functioning cells that exhibit uncontrolled growth, anticancer drugs indiscriminately impact the growth of normal non-proliferative cells along with inhibiting cancer cell growth. This poor selectivity of common chemotherapeutic drugs imparts serious side effects on normal tissues such as bone marrow, hair follicles and the gastrointestinal tract [5]. To quote some examples: Carboplatin or carboplatin in conjunction with other chemotherapeutic agents have been known to induce dose-dependent hematotoxicity such as neutropenia and thrombocytopenia. Dermatological effects, specifically keratitis, are common skin reactions arising from chlorambucil administration. Also, dose-limiting glomerular and tubular dysfunction, nuclear pallor in distal nephron and mitochondrial swelling may be caused by renal accumulation of cisplatin by a membrane transport assisted process after continuous and long-term exposure [6]. Acute cardiotoxicity that may include arrhythmias, acute heart failure, inflammatory responses such as pericarditis and myocarditis and other related symptoms including apoptosis due to formation of free radicals, and cardiomyocyte dysfunction are known to be caused by accumulation of Anthracyclines, specifically doxorubicin [7]. In addition to acute toxicity, chronic cardiotoxicity such as left ventricular dysfunction is also related to anthracyclines [7]. For breast cancer treatments, emesis, neutropenia and alopecia are common symptoms of 5-fluorouracil (CMF) cyclophosphamide and methotrexate regimen [8].

Besides the above-mentioned significant examples of severe side effects of chemotherapeutic agents, there are other side effects that are not as potent but do severely limit quality of life and may lead to premature discontinuation of chemotherapy. Dermatologic reactions are most prevalent between them [9]. Common skin related adverse effects include hyperpigmentation, dryness and rash. Other common skin reactions such as erythema and swelling are generally associated with antimetabolite drugs such as CMF and capecitabine [9]. Relatively new anticancer drugs e.g., epidermal growth factor receptor (EGFR) inhibitors noticeably cause follicular rash (e.g., papulopustular rash) and dryness that can then lead to infections such as pruritis [9]. Besides skin, other common side effects are observed on mucosal membranes where conditions such as toxic epidermic necrolysis and Steven Johnson Syndrome (SJS) are caused by other drugs e.g., busulfan, chlorambucil, cyclophosphamide and procarbazine [9].

**Figure 1:**
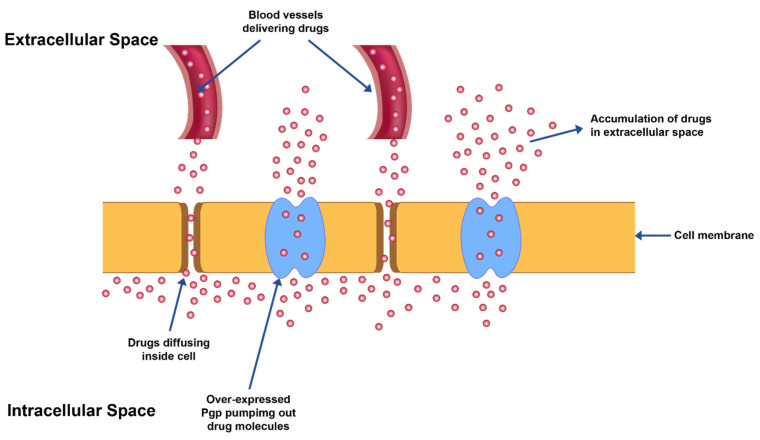
MDR exhibited by overexpression of Pgp transporter proteins leading to efflux of drug from cancer cells.

In furtherance to the adverse effects mentioned above, owing to poor selectivity/non-specificity of chemotherapeutic agents against cancer cells, the other significant limitation is the advancement of ‘multi-drug resistance’ (MDR) after prolonged exposure of drugs (Figure 1). Cancerous cells may grow resistance against a single chemotherapeutic agent or a combination of agents with an analogous mechanism of action but may develop into cross-resistance against other agents with differing mechanism of actions and/or targets. This transformation to cross-resistance against other therapeutic agents is called ‘multi-drug resistance’ (MDR). It is due to the development of MDR that heterogeneous cancer cells grow even in the presence of chemotherapeutic drugs. The development of this drug tolerance is manifested in cancer cells either as modification in a potential drug target or as augmentation of cell survival mechanisms such as DNA repair, changes in apoptotic cycles due to changes in ceramide levels, ineffective tumor suppressor protein (p53) or activation of cytochrome oxidases which is critical for cellular respiration [10].

MDR also leads to over-expression of ATP binding cassette-based efflux transporters which in turn reduce the drug levels in the intracellular space to suboptimal levels in the cells (Figure 1).

The severity of side effects caused by chemotherapy, as well as the MDR phenomenon combined with the narrow therapeutic index of anticancer drugs, severely limits the therapeutic efficacy of chemotherapy. Furthermore, severity of side effects necessitates dose reductions of the anticancer agent which eventually leads to inefficient therapeutic outcomes and potential metastasis.

### 2.3. Radiotherapy

Radiotherapy is another prominent anticancer therapy and is characterized by the use of high-energy radiation for the treatment of cancer. The wide application of radiotherapy varies from eliminating tumor to reducing tumor size. One way in which radiotherapy differs from chemotherapy is that the adverse effects of radiotherapy are localized in nature (in proximity to the radiated area) as opposed to systemic adverse effects manifested by chemotherapy. The side effects of radiation therapy can be classified either as early or late effects. While early effects are reversible, late effects have propensity to be irreversible and aggravate with time. The more involved late effects are facilitated by stromal, parenchymal, inflammatory and endothelial cells.

Early adverse effects are largely skin reactions such as desquamation and erythema. On the other hand, late effects consist of conditions for example radiation-induced neuron and blood vessel injury, atrophy and fibrosis. Fibrosis is a condition defined by buildup of excessive collagen and extracellular matrix in and around radiated tissues. The early phase of fibrosis is characterized by activation of cytokine cascades which yields tumor-necrosis factor-α (TNFα), interleukins 1 and 6 and other growth factors in much similarity to the wound healing process [11]. In contrast to a regular wound healing process, however, which is a short-term process, fibrotic factor TNF β is downregulated by TNFα and connective tissue growth factor (CTGF); the fibrogenesis in tissues continues for years, resulting in fibrosis of tissues [11].

## 3. Targeted Drug Delivery Systems and Their Limitations

As mentioned earlier, chemotherapy finds its limitation in being indiscriminate, non-specific in its mechanism of action and development of MDR. The sum of these effects renders chemotherapy damaging to normal dividing cells and thus, causes multiple side effects and, over prolonged exposure, becomes less effective to the tumor due to the development of MDR. Notably, less than 10% of an anticancer drug reaches its target tumor tissue [12]. In addition, radiotherapy primarily is localized in its effect and may lead to fibrosis in some cases. Targeted drug delivery systems, on the other hand, specifically target cancer cells while sparing normal cells. Most of the targeted nano drug delivery systems developed in the last few decades include liposomes, antibodies, Immunoconjugates, Immunotoxins, and polymer conjugates among others. Some of these delivery systems are discussed in this review with greater emphasis on liposomal drug delivery systems. It is important to note that these delivery systems have different mechanical and physicochemical properties than individual constituents’ lipids, allowing these microstructures to incorporate highly insoluble and/or unstable drugs that can be delivered in designated dosages to the target site.

Drug delivery approaches designed for targeting tumors can be largely classified into two main types: Active and Passive targeting approaches.

Some recent examples of anticancer liposomal drug delivery systems and their targeting mechanisms is provided in Table 1.

### 3.1. Active Tumor Targeting Approach

Active targeting at the molecular level discriminates between normal and cancerous cells by acting upon their morphological, phenotypic, and biochemical differences. A common active targeting approach involves ligand–receptor or antigen–antibody binding interactions to locally deliver cytotoxic drugs to tumor cells. The precise drug delivery mechanism in most instances is via receptor-mediated endocytosis after interaction of a drug or a drug carrier molecule with a specific antigen/receptor. The cytotoxic agents are associated with tumor specific ligands either directly via a carrier molecule.

**Figure 2:**
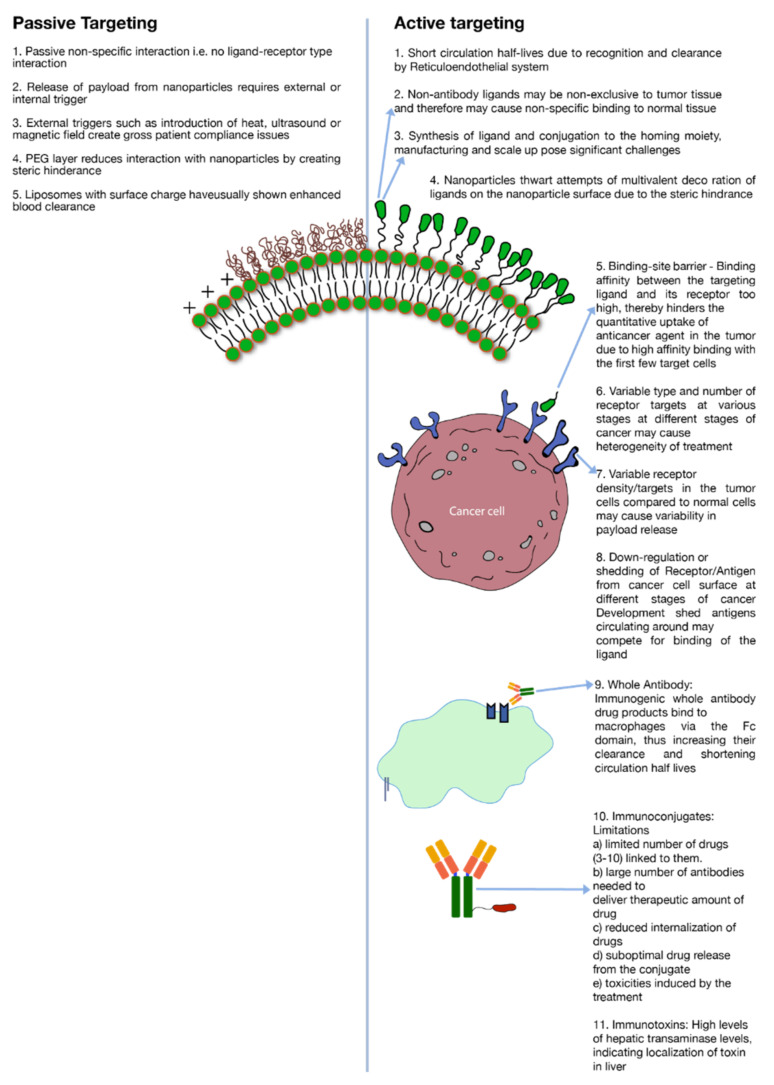
Limitations of active and passive targeting.

A major limitation to active targeting, however, is antigen heterogeneity. As stated earlier, different kinds of cancers or even same kind of cancer expresses different biochemical and morphological characteristics at different stages of their development which creates heterogeneity in the antigen expression (Figure 2). Receptor density is another important criterion to consider in active targeting. For a discriminatory effect, it is critical that the number of receptors are over-expressed in the cancer cells as compared to normal healthy cells (Figure 2). To illustrate this point, for enhanced breast cancer efficacy a receptor concentration of 10^5^ per cell of the tyrosine-protein kinase receptor (CD340) was deemed essential [5]. Likewise, a concentration of up to 10^5^ per cell of CD19 antigens was required for effective targeting of B cells by anti-CD19 antibody conjugated to liposomes [68]. Besides receptor density, during the development of cancer, shedding of antigens or their down-regulation may severely alter receptor concentration on the cell surface. Moreover, shed antigens may compete for interaction with an administered ligand, which is directed towards antigens attached to the cancer cell surface. Depending on the level of shedding, this phenomenon might impact the level of cytotoxic agent internalization to the cancer cells (Figure 2) [5]. Moreover, if the ligand–receptor binding avidity is very strong then it will impede the penetration depth of the anticancer agent in the tumor tissue due to ‘binding-site barrier’ where conjugated drugs are strongly bound by the first few receptor targets in the tumor tissue (Figure 2) [5]. As an example, it is reported that SK-OV-3 ovarian cancer targeting by single-chain fragment variable (SCFv) was dictated by the binding avidity of the SCFv antibody against the human epidermal growth factor 2 (HER2) receptors [69]. Binding affinity of a mutant-type Fv molecule over and above 10^−9^ M leveled off the distribution of Fv in the targeted tissue [69].

#### 3.1.1. Antibody and Antibody Fragments

Cancer cell targeting strategies involving antibodies have employed either whole antibodies or their fragments. While whole (intact) antibodies are generally considered more stable, they possess multiple binding sites. The presence of these sites makes them vulnerable to recognition by white blood cells in the body. A common interaction and, therefore, their clearance mechanism is the binding of their Fc domain with macrophages. (Figure 2) [5]. This binding triggers a cascade of immunogenic reactions which leads to rapid clearance of antibodies from the blood circulation. Mechanisms have been developed to modify the antibodies to yield more humanized or chimeric antibodies that invoke a less intense immune reaction; however, the development and manufacturing of such systems have proven to be challenging. In addition, when whole antibodies are conjugated to nano-carriers (liposomes, nanoparticles etc.) the ability to impart multivalent decoration is severely restricted due to the steric hindrance (Figure 2).

As a potential solution, antibody fragments were introduced that have specific binding sites such as Fab, Fv or ScFv but are relatively less stable as compared to parent antibodies. Also, these fragments carry less binding avidity due to their monovalent binding sites. Furthermore, attempts to use non-antibody peptides/proteins such as RGD (Arg-Gly-Asp), folate and transferrin have yielded non-specific results due to lack of disparity or receptor density in expression of their targets among tumor and normal tissues (Figure 2) [5].

Another alternate domain that is fast catching up is targeting using nanobodies. Nanobodies are naturally found in camel, llama or whales and are more comparatively stable than whole antibodies but they still have to find their clinical relevance.

#### 3.1.2. Immunotoxins and Immunoconjugates

Immunotoxins are either similar to antibodies or are antibodies conjugated to toxins to render them cytotoxic. However, the whole conjugated molecule induces moderate to severe adverse effects which limits their use. A couple of severe side effects include high internalization in liver indicated by higher expression of liver transaminase (Figure 2). Other notable side effects of immunotoxin therapy are vascular leak syndrome (VLS) and influenza-like symptoms [5]. Also, blocked ricin (toxin) conjugated to anti-B4 antibody has demonstrated anti-ricin and human anti-mouse antibody responses [5].

Immunoconjugates, on the other hand, are close analogues of Immunotoxins where instead of a toxin, an anticancer drug is conjugated to an antibody or protein. Antibody-drug conjugates (ADC’s) also fall under this category. As a conjugate, the cytotoxic effect is imparted by the cytotoxic drug while the targeting is driven by the associated antibody or protein. There are several constraints of using immunoconjugates as a potent tool against tumor. Prominent among these are: (a) limited number of cytotoxic agents that can be conjugated to anchoring molecules without severely impacting its binding avidity towards target antigen. On an average, 3–10 molecules of cytotoxic drug are known to conjugated to the anchoring antibody [5] (Figure 2). (b) Due to limited number of drugs conjugated to each antibody, a high number of antibodies are required to deliver therapeutic levels of the drug. (c) Poor localization of actives; (d) suboptimal drug release from the conjugate; (e) ADC related toxicities e.g., gastro-intestinal (GI) toxicities caused by SGN-15 immunoconjugate [5,70] (Figure 2).

#### 3.1.3. Immunoliposomes

Immunoliposomes are liposomes that carry targeting or anchoring ligands/antibodies on their surface. The conjugation of targeting ligands/antibodies is achieved either by bioconjugation with exposed sulfhydryl groups attained after di-sulfide bond reduction [71] or through lysine functionalization using 2-Iminothiolane [72,73], N-succinimidyl 3-(2-pyridyldithio)propionate (SPDP) [74] or N-Succinimidyl-S-acetylthioacetate (SATA) [75]. Click chemistry of azide functionalized phospholipids with cyclooctyne modified antibodies is a most recent example [76].

Immunoliposomes usually have intravascular and extravascular targets. Intravascular targets are considered more accessible for intravascularly administered immunoliposomes. Anti-VEGFR2 and anti-VEGFR3 Dox loaded immunoliposomes are common examples that have resulted in greater reduction in tumor mass in animal studies using antibodies against vascular endothelial growth factor receptors for targeting tumor-associated neovascular endothelial cells [77]. Other immunoliposome targets include brain, uterus, red blood cells, and T lymphocytes among others. Anti-Transferrin receptor (TfR) immunoliposome is one example where anti-amyloid-β antibodies were targeted across the brain–blood barrier [78]. Anti-vascular cell adhesion molecule (anti-VCAM), anti-TfR and anti-intercellular adhesion molecule (anti-ICAM) immunoliposomes were screened for optimizing blood to brain drug delivery ratios [76]. Anti-oxytocin receptor (OTR) immunoliposomes were studied for drug delivery to the uterus [79]. Additionally, Moles E. et.al investigated anti-Glycophorin A (GPA) immunoliposomes for antimalarial drug delivery to malaria-parasitized RBCs [80,81]. Similarly, Ramana et al., attempted anti-HIV drugs loaded anti-CD4 immunoliposomes delivery to T lymphocytes [82].

Multiple immunoliposome targets have been discussed above and are also shown in Table 1. It is important to note that there are some fundamental challenges associated with immunoliposomes.

Although, immunoliposomes can carry a large payload of drug molecules in their lipid bilayer or their aqueous interior, and, therefore, have high drug to antibody ratio, on the flipside, immunoliposomes carry only limited number of antibody molecules on their surface due to steric hinderance. Also, the bulky and complex structure of these systems triggers an immunological response and, therefore, enhances their systemic clearance (Figure 2). Circulating plasma proteins form protein corona upon exposure of liposomes, thereby triggering opsonization by complement proteins. Immunoliposomes, therefore, are subsequently cleared from blood circulation by reticuloendothelial system (RES) in liver and spleen [83]. Furthermore, immunoliposomes need to be optimized to contain the effects of heterogenous tumor properties, else the efficacy may vary depending upon several histological and microenvironmental factors as mentioned previously. Potentially, Immunoliposomes can be decorated with two different antibody fragments to target multiple epitopes on tumor cells, or even different cells population on the tumor tissue [84]. However, the receptor/antigen density and the affinity of the antibody for a specific antigen or the ‘binding-site barrier’ issue (Figure 2) may still pose a barrier which may lead to poor tumor penetration and poor efficacy against cells with down-regulation of target antigens. Designing immunoliposomes are becoming increasingly valuable and highly challenging with the evolution of new therapeutic modalities such as like siRNA and mRNA etc., as payloads [85].

#### 3.1.4. Manufacturing and Clinical Challenges of Active Targeting

A significant manufacturing challenge associated with active targeting, specifically immunoliposomes, is the scale up of nanomaterial manufacturing process. The issue is two pronged, firstly the large-scale manufacture of the constituent lipid–ligand conjugate (Figure 2) and secondly, the large-scale preparation of liposomes using the constituent lipids with consistent particle size distribution and lamellarity (Table 2). The conjugation of lipid–ligand conjugate is usually a multi-step synthesis process that involves use of organic solvents. This increases complexity and cost of production during cGMP (current-Good Manufacturing Practices) scale up of the conjugate and subsequent formulation preparation. It is important to note that the functional stability of the conjugate is important during various processing conditions as the incorporation of nanoconjugate alters the chemical makeup of the nano-formulation and leads to uncertainty in biodistribution, pharmacokinetic, and pharmacodynamic profiles [86]. Another problem is the differences in the heterogeneity in the cancer cell receptor expressions between small animals (rodents, rabbits) to humans. The optimization of the drug product to maximize its interaction with receptors on the cancer cell surface depends on the correlation of human vs. animal data and hence the translation of preclinical study results into clinical studies.

### 3.2. Passive Tumor Targeting Approach

**Figure 3:**
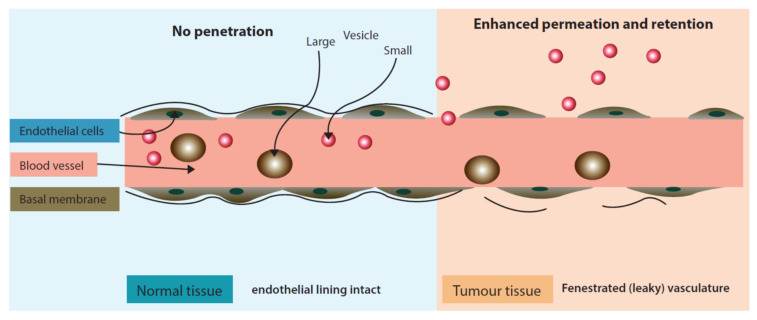
‘Enhanced Permeation and Retention’ effect exhibiting enhanced permeability of liposomes in inter-tumoral space.

Passive targeting approach is distinct as it does not utilize a ligand/receptor or antibody/antigen interaction but rather exploits physiological characteristics of the tumor micro-environment. Passive targeting largely exploits the ‘Enhanced Permeation and Retention’ effect (Figure 3) for the localization of drugs in the tumor tissue. The enhanced permeation (localization) of nano drug delivery systems in the tumor occurs due to fenestrated tumor blood vasculature. Once permeated in the tumor environment nano drug delivery systems are retained at the target site due to poor lymphatic drainage.

It is worthwhile to note that while passive targeting eliminates some of the issues of active targeting (e.g., antigen heterogeneity, receptor density, etc.) it brings its own limitations to the cancer therapy. To cite a few examples: low drug release at target site, high systemic clearance of surface charged nano drug delivery systems (Figure 2) and the need for either external stimuli i.e., heat, light and/magnetic field at the tumor site or endogenous stimuli i.e., pH, hypoxia etc., to trigger drug release (Figure 2). Only liposomal drug delivery systems and their external drug release trigger mechanisms are discussed in detail in this review.

#### 3.2.1. Traditional Liposomes

Historically, very early liposomes i.e., traditional liposomes introduced in the 1960s were devoid of any biocompatible polymer coating on their surface. Upon administration to systemic circulation, traditional liposomes trigger immune response by mononuclear phagocyte system (MPS). This activation of the immune system is indeed exploited in treatment of some bacterial/fungal infections of the immune system. A standard example is liposomal amphotericin B which is targeted to fungus-infected macrophages. This liposomal drug was primary line of treatment during recent SARS-COV-2 related fungal infections in India [87]. Beyond targeting the MPS system, traditional liposomes find little significance in targeting tumor cells due to rapid recognition and clearance by macrophages [88,89].

At the molecular level, cationic liposomes are more prone to recognition by MPS because of their affinity towards negatively charged serum proteins. Serum protein bound liposomes have tendencies to trigger the MPS system owing to their bigger size. As an example, cationic liposomes prepared with equimolar mixture of cationic lipid 1,2-dioleyl-3-N,N,N-trimethylaminopropane chloride (DOTMA) and neutral lipids i.e., DOPE or DOPC have propensity towards serum protein binding as depicted by their protein binding (PB) value 500g protein/mol or higher [90]. In a similar study, keeping DOTMA up to half of the total lipid composition of liposomes resulted in very strong plasma protein interactions that triggered formation of clots [91]. Moreover, liposome formulations prepared with another cationic lipid i.e., N-N-dioleoyl-N,N-dimethylammonium chloride (DODAC) and DOPE showed a very high PB value of 800g protein/mol and subsequently poor apparent half-life of only a few minutes [90]. Cationic liposomes even higher PB values of up to 1100 g proteins/mol have also been reported [90,92].

To enhance the circulation half-lives of liposomes various approaches have been implemented. One such approach is to alter the surface charge composition of liposomes by addition of a negatively charge lipid phosphatidyl inositol to the liposomal formulation which stabilizes the liposomes in vivo [93]. Liposomes prepared with hydrogenated phosphatidyl inositol/phosphatidyl choline/cholesterol (HPI/HPC/CH) exhibited an apparent half-life of 15.5 h of encapsulated doxorubicin as compared to 1 h of traditional liposomes prepared with egg-originated phosphatidyl glycerol (PG), phosphatidyl choline (PC) and cholesterol [93].

#### 3.2.2. Stealth Liposome

Since high blood clearance of traditional liposomes was a major challenge, as a breakthrough to this problem, liposomes grafted with a biocompatible polymer that could evade immune recognition were introduced. Polyethylene Glycol (PEG) is one such example of a biocompatible polymer. Presence of the PEG–lipid conjugate allows for formation of a sterically stabilized hydrophilic aqueous shell that renders the liposomal system evasive to the immune system. Due to the steric stabilization imparted by the PEG layer, serum protein binding blood clearance of the liposomes is greatly reduced. The PEG coated liposomes are, therefore, called ‘Stealth Liposomes’ (SL) due to evasive nature of these systems.

**Figure 4:**
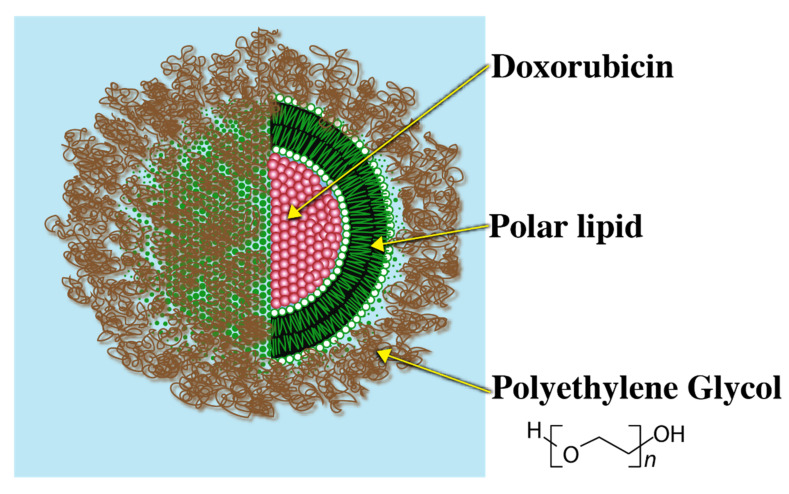
Doxil coated with Polyethylene Glycol.

A commercially available relevant example of a stealth liposome (SL) is ‘Doxil’ (Figure 4).

An example of significant improvement in the performance of a drug using SL technology is Epirubicin. Administration of un-encapsulated Epirubicin causes rapid blood clearance of the drug yielding a very short half-life of only 14 min. On the contrary, the half-life of an encapsulated form of the drug was significantly higher i.e., 18 h [93]. This improvement was also reflected in the bioavailability of the drug where the AUC of encapsulated form showed more than 200X increase than the un-encapsulated form [93].

Other important marker of the immune system avoidance is the measure of PB. It has been reported that addition of a PEG layer markedly lowers the PB value [90,94]. To cite a few examples, traditional liposomes composed of egg-PC/CH/1,2-dioleoyl-sn-glycero-3-phosphate (EPC/CH/DOPA) and DSPC/CH in a mol ratio of 35:45:20 and 55:45, respectively, had PB values of 46 and 19, respectively. However, upon the addition of 5% of a PEGylated lipid i.e., 1, 2-Distearoyl-sn-glycero-3-phosphoethanolamine-Poly(ethylene glycol) (DSPE-PEG), to the respective formulations, reduced the PB values to 25 and 7, respectively [90]. Similarly, blood cell binding of glass coated DPPE/DSPE-PEG liposomes significantly reduced as the level of PEGylated lipid DSPE-PEG in the liposomes was raised from 0 to 1 mol% [94]. The rate of cell binding, however, reduced with higher levels of DSPE-PEG added to the liposome [94].

Although implementation of PEGylated lipids has largely improved the circulation half-life and localization of liposomes in target tissues, there are major drawbacks associated with the SL technology. Since the steric hinderance imparted by PEG limits liposomal binding to immune cells, equivalently, it also limits the binding and subsequent internalization to tumor cells once liposomes have extravasated to tumor environment (Figure 2). It was reported that a Te parameter i.e., AUC tumor/AUC plasma ratio was nearly three-fold lower for PEGylated liposomes (DSPC/CH/DSPE-PEG) as compared to traditional liposomes DSPC)/CH in C26 tumor bearing mice [95]. Similarly, in the Lewis lung model study using DSPC/CH and DSPC/CH/PEG-PE, Te values for the PEGylated liposomes were approximately half of the non-PEGylated liposomes [96].

Due to reduced internalization of liposomes by tumor cells, once reaching the tumor tissue, the drug release largely relies on passive diffusion of drug to extra-liposomal space, which is a slow process and leading to sub-optimal levels of an anticancer drug in tumor.

#### 3.2.3. Requirements of Stimuli Induced Drug Release

To significantly increase drug release from liposomes accumulated in a tumoral space, endogenous triggers or external triggering mechanisms have been envisioned. Endogenous triggers in the tumor micro-environment are acidic pH, hypoxia, enzymatic degradation, etc. The major limitations of endogenous triggers are chemical instability of pH sensitive lipids [4,97], poor hypoxic heterogeneity in tumors [98,99] and less reliable enzyme heterogeneity in tumors [100]. Barring enzyme heterogeneity issue in the tumors, peptide-based supramolecular assembly/disassembly provides an interesting trigger mechanism that can retain the drug cargo in blood circulation and release upon enzymatic hydrolysis in the tumor environment [101,102]. One such study was performed by Kalafatovic et al. [103] where a peptide micelle was converted to fibrillar nanostructures to trigger drug release. The trigger for such conversions is change in the hydrophilic–lipophilic balance triggered by enzymatic hydrolysis. In an interesting approach, to avoid heterogeneity in tumor environment by utilizing all the above endogenous triggers (i.e., pH, redox potential and endogenous proteinase concentration), Zhang et al. [104] developed a protein based nanospheres for triggered release of encapsulated Chlorin e6.

External triggers, on the other hand, complicate the therapy by requiring external clinical intervention and thereby creating gross patient incompliance. In this review, external trigger mechanisms such as application of ultrasound, light and temperature to trigger drug release are discussed in detail.

##### Ultrasound Induced Triggered Release

Ultrasound (US) are mechanical longitudinal waves propagate due to pressure changes in the medium with a periodic vibration of frequencies higher than the human audible range of 20 kHz [105]. Drug release by US is due to different mechanisms such as cavitation, acoustics and hyperthermia [106]. Cavitation and acoustics related mechanisms are more widely used and discussed here. VanOsdol and colleagues utilized ultrasonic cavitation for DXR drug release by incorporating perfluoropentane gas (PFP5) in liposomes [107]. They observed that microbubble formation by using PFP5 was able to increase the drug concentration to target tissues to 1.4 times upon the use of high intensity focused ultrasound (HIFU). Another prominent drug release mechanism by acoustics and is well demonstrated by acoustically active liposomes (AAL). AAL’s possess air pockets that may expand upon pressure change and upon exposure to US radiation. The expansion of air pockets leads disruption of liposomal membrane and, therefore, release of encapsulated contents. A significant benefit of this trigger mechanism is that it is a non-invasive technique which can be controlled. Besides triggering drug release from liposomes, this technique can also alter the permeability of cell membrane [108,109]. A prominent example of AAL’s is calcein-loaded liposomes prepared from EPC/DPPE/1,2-Dipalmitoyl-sn-glycero-3-phosphoglycerol (DPPG)/CH at a molar ratio of 69:8:8:15 [108]. The calcein release was shown to be well controlled, however, encapsulation efficiency of these liposomes was very low (≤ 20%) [108]. Additionally, such systems have not been tested for encapsulation and the release of hydrophobic drugs that localize in the lipid domains of the liposomes rather than aqueous interiors of liposomes.

##### Light Induced Triggered Release

**Figure 5:**
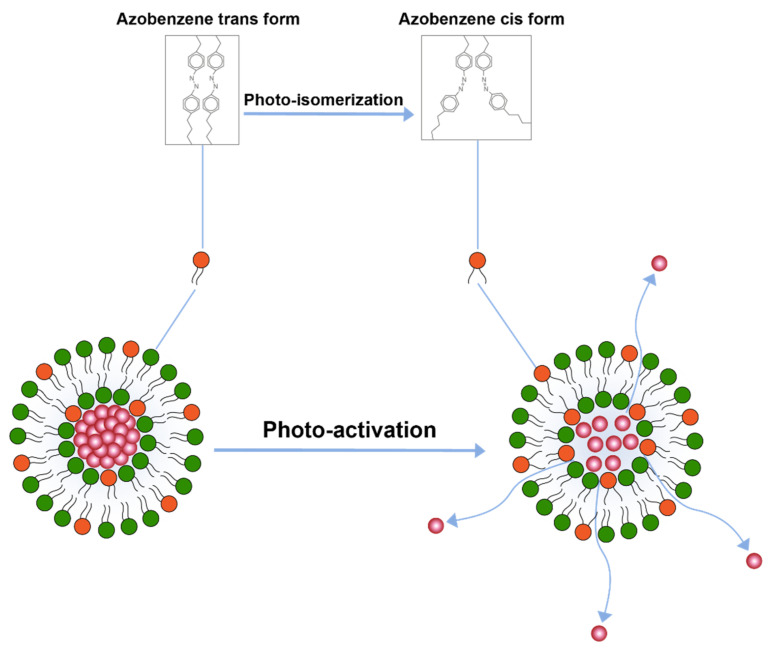
Drug release from liposomes by photo-isomerization of lipids.

Another important trigger is the application of light for drug release. A study conducted by Leung et al. [110] explored triggered release by light induced chemical changes in the lipid constituents of the liposomes. Broadly, these changes include photo-isomerization, photo-cleavage or photo-polymerization of photo-sensitive lipid constituents of the liposome membrane. A majority of photo-sensitive liposomes incorporate isomerizable lipids that can convert form one isomeric form to the other upon light activation. A prominent example of these liposome includes the ones that are prepared by azobenzene lipid. Azobenzene can isomerize to cis form upon exposure to UV-light and converts back to the transform upon exposure to visible (blue) light [110] (Figure 5).

This isomerization of lipids disrupts membrane structure and releases encapsulated contents. Besides azobenzene, light sensitive liposomes composed of retinoyl-phospholipids [111] and spiropyran, which converts to merocyanine at lower visible 365 nm, have also been tested [112]. The major challenge with photo-isomerization is that the wavelengths required to photo-isomerize the photosensitive lipids fall in lower visible spectrum which have poor penetration depth in the body.

**Figure 6:**
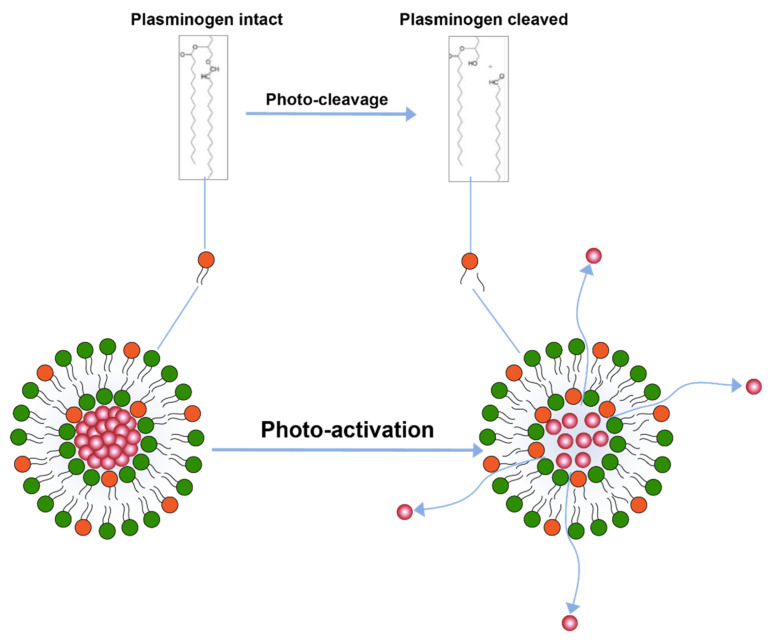
Drug release from liposomes by photo-cleavage of lipids.

Another trigger mechanism that uses light activation is inclusion of photo-cleavable lipid constituents in the liposomal membrane [110] (Figure 6). Photo-cleavable lipids essentially break-down upon exposure to lipid and thence disrupt the membrane structure. The cleavage upon light exposure causes changes in the hydrophilicity of the lipid constituents which are, therefore, not able to retain membrane structure and allow for drug release. Similar use of photocleavable lipids derived from plasmalogen have also been reported [113]. Effect of photo-cleavage may be assisted by use of photosensitizers molecules such as tin octabutoxyphthalocyanine, zinc phthalocyanine, or bacteriochlorophyll a. [114].

The photosensitizer molecules, however, result in the formation of reactive oxygen species in the body which may compromise patient safety [4].

Furthermore, dithiane-based lipids have also been reported to increase the drug release from liposomes [115,116]. In an interesting report, a synthetic DOPE-based photocleavable lipid NVOC-DOPE was transformed to DOPE upon exposure to xenon lamp, causing subsequent liposome membrane disruption [117]. Yavlovich, et al., have also investigated the inclusion of 2-nitrobenzyl lipid derivate of PC, named NB-PC in liposomes for the photo-cleavage triggered release. Nile red was found to increase its release relative to the concentration of NB-PC in the liposome upon UV irradiation at 350 nm [118].

**Figure 7:**
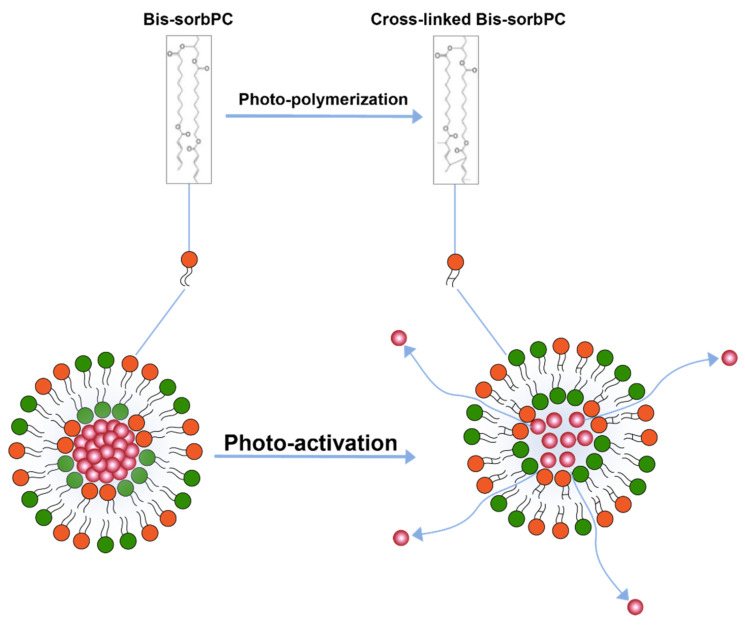
Drug release from liposomes by photo-polymerization of lipids.

Photo-polymerization is another light triggered drug release mechanism [110,111] (Figure 7) where polymerization of lipids upon activation by light causes pairing of photo-polymerizable lipids on the liposome surface creating loose pockets on liposome surface from where drugs may escape the liposome interior. Formation of these loose pockets on liposome surface is also aptly illustrated by the polymerization of a lipid 1,2-bis [10-(2′,4′-hexadienoyloxy)-decanoyl]-sn-phosphatidylcholine (bis-SorbPC) after upon UV exposure which resulted in more than 100X increase overall release of a fluorescent molecule [111,119]. Similarly, Yavlovich and co-workers demonstrated much higher MCF-7 breast cancer cell inhibition using 514 nm light exposure after delivering by doxorubicin loaded in liposome containing photopolymerizable lipid 1,2-bis (tricosa-10,12-diynoyl) sn-glycer-3-phosphocholine (DC 8,9 PC) [120].

The UV wavelength required to trigger the release may be tuned by inclusion of photosensitizing dyes such as 1,1’-dioctadecyl-3,3,3’,3’- tetramethylindocarbocyanine iodide in liposomes towards the higher UV wavelengths that are considered biologically safe [111].

Photo-polymerization is an intriguing concept, however, there are a few limitations, such as the stability of polymerizable lipids in aqueous suspensions have not been tested yet [111], and more importantly, the penetration depth of UV light in human subjects remains a challenge.

Additionally, in an interesting photo-oxidation approach using Bacteriochlorophyll/diplasmenylcholine (Bchl:DPPlsC) liposomes, it was reported that photo-oxidation induced drug release is severely impacted by hypoxic (low Po2) tumor environment [121]. Poor photo-oxidation leads to development of physiologically favorable atmosphere for growth of non-apoptotic cells [121]. Other such approach of utilizing photo-oxidation include the addition of photochemical agents such as Porphyrin-phospholipid (POP), talaporfin sodium (TPS) and Indocyanine green and octadecylamine (ICG-ODA) in combination of oxidation sensitive lipids containing liposomes such as DOPC, cholesterol and 1-(1z-octadecenyl) -2-oleoyl-sn-glycero-3-phosphocholine (PLsPC) and have reported in better cytotoxicity upon photo-exposure conditions. All the photo-oxidative approaches, nevertheless, require external stimuli imparted by near infra-red application [122,123].

##### Hyperthermia Induced Triggered Release

**Figure 8:**
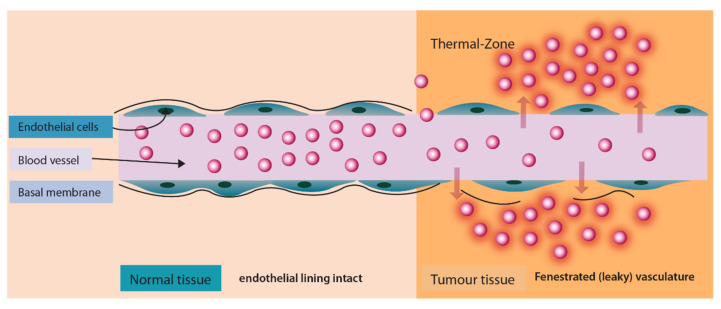
Enhanced liposomal extravasation and drug release at tumor site upon application of heat.

A different technique to trigger drug release after localization of liposomes in the tumor tissue is by application of heat. This approach is significant as it provides a variety of advantages. Firstly, the application of heat enhances the blood vessel permeability at the tumor site which causes enhanced extravasation of liposomes (Figure 8). Secondly, it triggers the drug release from liposomes in the vicinity of the tumor (Figure 8) and thirdly, it enhances the blood flow and increases the drug uptake. One such drug release mechanism was implemented for the release of Neomycin [124].

**Figure 9:**
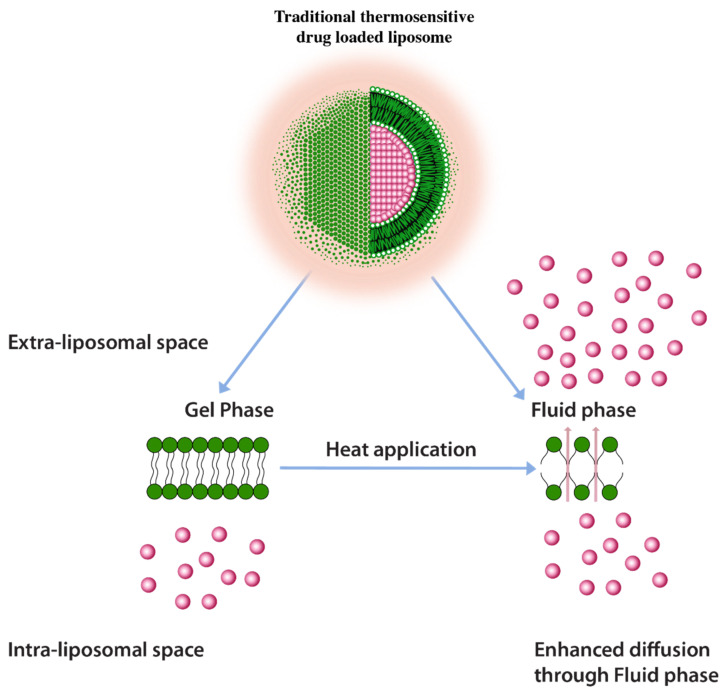
Traditional thermosensitive liposomes showing gel to fluid phase transition upon application of heat.

A common approach to enhance drug release using thermos-sensitive liposomes is the inclusion of lower melting lipids in the liposome composition. In some examples, the application of heat induces trans to a gauche conformational change of the constituent lipids at their transition temperature [125]. This transitions the gel phase of the lipid bilayer to the fluid phase and triggers the rate of drug diffusion and hence the drug release enhancement (Figure 9) [125]. It is, however, important to note that a homogenous or nearly homogenous composition of bilayer by inclusion of lower melting lipids such as 1,2-Dipalmitoyl-sn-Glycero-3-Phosphatidylcholine (DPPC) (MP = 41 °C) have resulted in a less than optimum drug release [125]. One challenge with having a low melting lipid is that the drug cargo is poorly retained during blood circulation due to a less rigid membrane structure. Furthermore, inclusion of lipids with different melting temperatures and carbon chain lengths results in the creation of membrane packing heterogeneity and, therefore, increases drug release. Such heterogeneity was depicted by the broadening of peaks in differential scanning calorimetry in liposomes composed of DSPC (MP = 53 °C) and DPPC lipids [126]. It should be, however, be noted that higher melting lipids would require higher temperatures to induce drug release (higher than 43 °C) which may cause necrosis to normal tissues in the vicinity of the tumor tissue [125]. According to one report necrosis on porcine muscle was initiated after 30 min. of heat application at 40–43 °C [127].

It, therefore, remains a challenge to fine tune the drug release at mild hyperthermia conditions (39–41 °C) for efficient drug cargo release.

**Figure 10:**
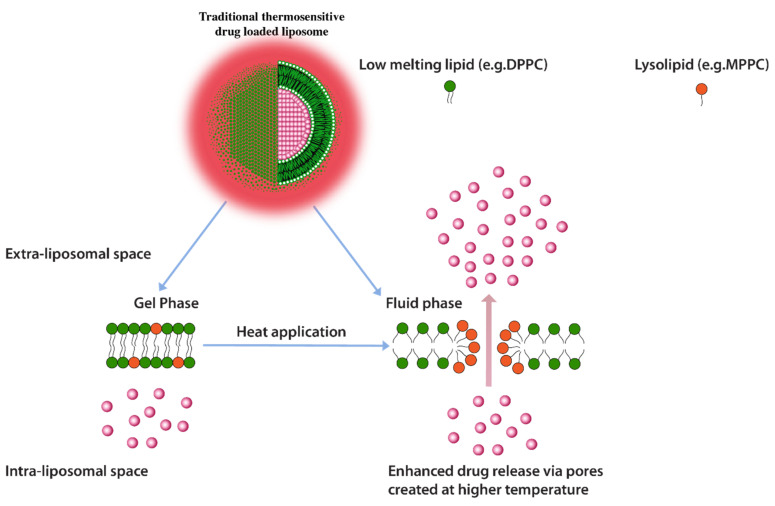
Traditional thermosensitive drug loaded liposomes showing formation of micellar pockets upon application of heat.

Another heat triggered drug release approach is inclusion of lyso-lipids as lipid constituents of thermo-sensitive liposomes. Lyso-lipids have a heavier head group with a single carbon chain and due to this typical structure, these lipids would characteristically form micellar structures. When incorporated into liposomes these lipids gain translational movement upon heating to their transition temperature and form early melting pockets on the membrane surface. These pockets arrange into micelle-like curved structure at these pockets which essentially create for enhanced drug release (Figure 10) [125]. Provided that lyso-lipids are included in appropriate ratios, it has been shown that these lipids effectively lower the phase transition temperatures required for triggered release. Liposomes prepared with 10 mol % of the Mono Palmitoyl Phosphatidyl Choline (MPPC) (lyso-lipid) lead to change in phase transition temperature to 39–40 °C from 43 °C and subsequently a very fast drug release i.e., nearly 50% after 20 s of exposure at 42 °C [128]. The rapid drug release, therefore, enables shorter heat exposure times which subsequently decreases the possibility of initiation of necrosis in normal tissue in the vicinity of the tumor [125]. However, the challenge with lyso-lipid liposomes is their in vivo stability, lyso-lipids impart instability to liposomal structure due to desorption of lyso-lipid while liposomes are in blood circulation. It has been reported that after 1 h of injection up to 70% of the lyso-lipids were desorbed from the liposome surface [129,130] and the amount of drug released recovered from liposomes mice plasma after 4 h of injection was significantly low [129]. Although, in a 2020 study, a phase clinical data of ThermoDox^®^ formulation of DPPC:MSPC:DSPE-PEG2000 (86:10:4 molar ratio) were announced but the in vivo stability in pigs suggest an estimated half-life of only 4.8 h. [131,132].

The instability of lyso-lipid based liposomes, therefore, significantly reduces the thermo-sensitive feature of these liposomes.

Another limitation with heat triggered release is that it can only impact tumors that are located close to the body surface as compared to deep seated tumors because of higher temperature requirements to reach the desired temperature differential. Attempts to insert electrodes (microwave and radiofrequency) to deep seated tumors remain impractical due to the insertion depth and invasive nature of these procedures [125]. As an alternative ultrasound with a controlled focal zone were developed but regardless, monitoring of temperature would still require temperature probes penetrated into the tumor environment [125].

Also, heat trigger imparted by microwave and radio wave applicators is limited due to the therapeutic depth of only up to 3 cm [128].

##### Triggered Release by Magnetic Field from Magnetic Liposomes

**Figure 11:**
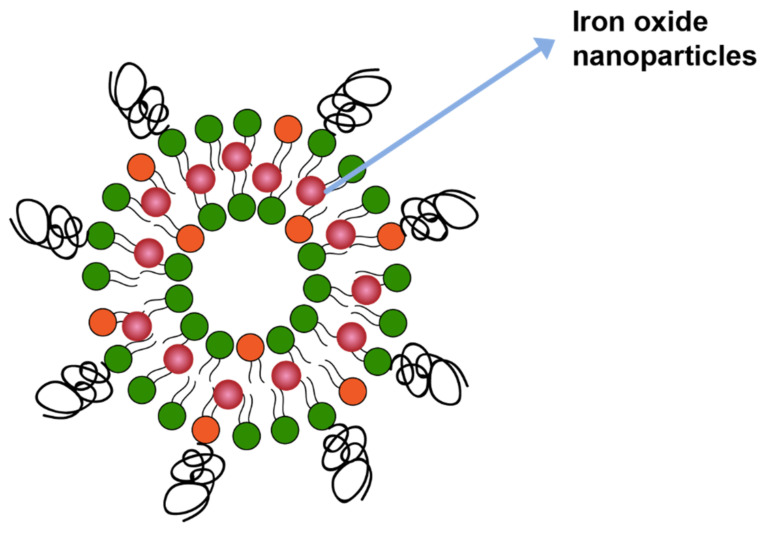
Iron oxide nanoparticles incorporated in lipid membrane.

Magnetic field is another mechanism that can trigger drug release from liposomes. In one study drug release was triggered by including iron oxide in the liposome membrane bilayer (Figure 11), which catalyzed local heating upon application of the magnetitic field. The local heating led to membrane disruption and, therefore, drug release [133].

Similarly, nearly 70 % of the drug Adriamycin was released when a ferromagnetic material was incorporated in liposome membrane bilayer at a ferro–colloid concentration of 1.2 mg Fe/mL [134].

In an interesting approach, liposomes were directed to tumor tissue by entrapping magnetite particles and then by applying a magnetic field on the target tissue [135].

In studies using Syrian male hamster limbs, under the influence of a study magnetic field in doxorubicin concentration increased 3X to 4X in tumor upon intravenous administration of magnetic liposomes [135]. Instead of having an externally placed magnetic field in the above examples, a non-magnetic alloy was rather placed directly in the tumor in another similar study using a limb tumor model [136]. A clear distinction between Adriamycin release with and without magnetic field was observed [136].

Additionally, a clear effect of the magnetic field on radiolabeled albumin loaded liposome was observed in rat models. Precisely, a samarium cobalt magnet was placed in the left kidney of rats and, therefore, showed a 25X increase in radioactivity in the left kidney compared to the right kidney which had no magnet [137].

In an interesting example, a 1.7X increase in cargo release at the tumor site was observed by using magnetic liposomes prepared with bacterial magnetic particles containing cis-diamminedichloroplatinum (II) as compared to magnetic liposomes prepared with synthetic magnetic materials [138].

Furthermore, in a unique dual targeting study, magnetic liposomes first targeted blood cells followed later magnetically directed to the brain for delivery of the cargo [139].

Although a variety of approaches have been employed, the application of the magnetic field has, until now, limited the use of magnetic liposomes due to gross patient incompliance.

### 3.3. Manufacturing and Clinical Challenges Common to Both Active and Passive Targeting

Besides requirements of triggered release and limited payload release at the site of action due to a multitude of reasons mentioned hitherto, active and passive targeting also presents manufacturing and clinical challenges (Table 2). The product related characteristics (in vitro drug release rate, particle size distribution, lamellarity, stability, drug encapsulation efficiency, etc.) obtained in a laboratory with a millimeter scale of the product should be reproducible when the product is scaled up in liters and should still maintain the same physicochemical properties and conform to the product release specifications. The manufacture, stability, degradation products, source and characterization of the lipidic components of the liposomes should be appropriately characterized prior to regulatory filing. In case of commercially available lipids, determining the positional specificity of acyl side chains is required and critical. In case of natural lipids (e.g., egg lecithin), the purification of the lipids is a challenge, and the composition of fatty acid requires robust analytical methods (Table 2). In addition, the analytical method should be qualified to distinguish and identify the lipid of interest in a mixture of lipids. Another crucial requirement is the determination of the amount of divalent cation and the counter ion content. The drug substance to lipid ratio at critical manufacturing unit operations is necessary and should be accurately and precisely determined to ensure consistent drug loading and drug release.

In addition, the approval of generic passively targeted liposomal products remains a challenge. The differences in the efficacy of lipodox which was launched a generic equivalent to Doxil has posed questions on the bioequivalence of the product in clinical trials [140]. The exact reason behind the difference although remains unknown.

Furthermore, the Pharmacokinetic/pharmacodynamic (PK/PD) profile of the liposome formulation is different from other conventional dosage forms. It is well known that the pharmacokinetic profile of liposomal amphotericin is different from the PK of amphotericin free drug [141]. It has been observed that the renal and fecal clearance of liposomal amphotericin is 10 times lower than the non-liposomal formulation [141]. The PK disposition of the drug depends on the PK of the liposomes which in turn depends on binding or fusion with plasma proteins and mononuclear phagocyte system (MPS), drug retention after dilution in the blood circulation and at low pH tumor environment [142,143,144]. Therefore, measuring the plasma concentration itself cannot be used to determined bioavailability. It should be noted that current FDA guidelines mandate that the bioanalytical method can determine both encapsulated and un-encapsulated drug content for product approval purposes. Early in the product development phase, radiolabeled liposomal products can be used for mass balance studies of plasma, urine and fecal samples to determine the PK profile. For liposomes not designed to be labeled, the quantitation of liposome accumulation in the tissue requires validated analytical methods that include tissue harvesting or organ isolation [145] and, therefore, pose a challenge in precise quantitative determination of liposomal accumulation and uptake by tumor tissues. This creates hurdles in translation from pre-clinical to clinical performance and, therefore, requires highly predictive models (Table 2).

In addition, formulation-based effects viz. size and method and level of drug loading on biodistribution of liposomes should also be considered, the release rate of drug using same lipid composition might provide different efficacy and safety profiles and mere more accumulation of liposomes at the tumor site cannot guarantee higher efficacy levels [146].

The rate of drug release and retention of the encapsulated drug depends on its physical state. When drug is precipitated inside the liposome carrier then the drug release is slower compared to when the drug is stored in solution form. The salt used to retain the drug also affects the encapsulation efficiency and drug release rate. Manganese sulfate has been known to be more effective in doxorubicin retention due to formation of complex rather than the ammonium sulfate salt [147].

As for the IV administration of liposomal formulation, the introduction of nanoparticles in the blood circulation stirs up stress reaction which is manifested in the form of activation related pseudo-allergy (CARPA) (Table 2). CARPA is caused as a result of nanoparticles entering the bloodstreams that are perceived as pathogenic organisms by the body. CARPA is managed by altering the rate of infusion, co-administering immune suppressants and employing less reactive infusion protocols [148,149,150]. CARPA has now gained attention by regulatory agencies and is perceived as safety risk in IV administration of liposomes [148]. European regulatory agency has now introduced CARPA test in pre-clinical test as a recommendation in development of generic liposomal formulations.

## 4. Need for Better Pre-Clinical and Clinical Strategies

Effectively translating preclinical research to clinical research is the need of the hour. Often, preclinical studies are conducted with a handful of liposome preparations; it is important to be able to perform high throughput screening of liposomes to comprehend biological and cellular interactions. Kelly et al. [151] have reported a number of cell interaction, toxicity and immune reactivity studies using high throughput methods. With the frequent use of well-developed high throughput techniques, correlation of biodistribution of liposomes and their PK/PD profile can be developed. The correlation of the PK/PD data, biodistribution and the efficacy data are of paramount importance. The determination of pharmacokinetic and tissue distribution profile is very critical for safety and efficacy determination of liposomes as the biodistribution is formulation specific and traditional bioequivalence studies may not interpret true biodistribution. The biodistribution profile of the liposomes is a key product of the formulation characteristics of a liposome. It has been known that the small size liposomes (≤ 100 nm) can reach deeper into tumor spheroid models [152]. It has also been known that the PEG coated liposomes have limited interaction with cancer cells compared to liposomes without any steric (Figure 2). This is especially helpful in segregating the effectiveness of the carrier in reaching the target and penetrating the tumor with the anticancer efficacy of the encapsulated drug.

The efficacy data experimentally determined should be extrapolated to humans using predictive computational/mathematical modeling. High throughput techniques and physiologically based pharmacokinetic modeling can also be developed for experiments involving in situ whole organs to understand biodistribution kinetics and predict PK parameters in humans.

A lot of work at the pre-clinical stage is performed using pharmacokinetic modeling tools, however, validated pharmacodynamic concentration vs. effect modeling systems need to be developed and implemented.

## 5. Conclusions

In this review, challenges and limitations associated with conventional anticancer therapies viz. chemotherapy, radiotherapy and cancer surgery were reviewed. Cancer targeting (active or passive targeting) as an alternate and break-through to some of the problems associated with the non-specificity of conventional therapies were discussed. While there is a certain appeal to using active targeting, as it is directed to the tumor, nonetheless antigen and receptor heterogeneity, immunogenicity, drug encapsulation level, etc., remain a problem. Moreover, passive targeting resolves some of the problems such as immunogenicity, these systems rely heavily on drug release triggering mechanisms (either external or endogenous stimuli). External stimuli were discussed in detail and are considered as either poorly effective or generate gross patient compliance issues. Lastly, there are manufacturing and clinical challenges associated with both active and passively targeted liposomes. In this regard, the need for robust analytical methods to determine biodistribution, PK and PD profile of liposomes was highlighted in addition to a critical gap between efficient preclinical to clinical efficacy predictive modeling.

We believe that while presenting the issues with current anticancer therapies, we also highlight the potential opportunities that will encourage further research in this area.

## Figures and Tables

**Table 1:** Some recent examples of anticancer liposomal drug delivery systems and their targeting mechanisms.

Active Ingredient/s	Trade/Brand Name	Liposome Composition	Active/Passive Targeting	Route of Administration	Indication	Ref.
Hwtp53 DNA	SGT-53	DOTAP/DOPE	Active (Anti-Transferrin scFv)	IV, in vivo, clinical	Solid tumors	[13,14,15]
Docetaxel prodrug	MM-310	Egg derived sphingomyelin/ CH	Active (Anti-Ephrin receptor A2)	IV, in vivo, clinical	Solid tumors	[16,17,18,19]
DOX	C225-ILs-dox	DSPC/CH/mPEG-DSPE	Active (Anti-EGFR Fab fragment from mAb C225 (cetuximab))	IV, in vivo, clinical	Glioblastoma	[15,19,20,21]
DOX	MM-302	HSPC/CH/DSPE-PEG	Active (Anti-HER2 antibody)	IV, in vivo, clinical	Breast cancer	[22,23]
Melanoma antigens + interferon-gamma	Lipovaxin-MM	POPC/Ni-3NTA-DTDA	Active (Single domain antibody (dAb) fragment (VH))	IV, in vivo, clinical	Malignant melanoma	[15,24]
RB94 plasmid DNA	SGT-94	DOTAP/DOPE	Active (Anti-Transferrin Antibody fragment (scFv))	IV, in vivo, clinical	Solid tumor	[15,25,26,27]
DOX	2B3-101	HSPC/CH/DSPE-PEG	Active (Glutathione ligand)	IV, in vivo, clinical	Active brain metastasis, meningeal carcinomatosis	[18,28,29]
Tetrandrine + vincristine	-	EPC/CH/DSPE-PEG 2000	Active (Transferrin ligand)	IV, in vivo in mice	Brain glioma	[19,30]
Bleomycin	-	DOPE/CH	Active (Folic acid ligand)	In vitro	Cervical and breast cancer cell lines	[19,31]
DOX	-	DOPE/DOPC/Lecithin	Active (Glycoprotein ligand)	IV, in vivo in mice	Mouse melanoma cells	[32]
ATRA	-	DPPC/CH/DSPE-mPEG2000	Passive	In vitro	Human thyroid carcinoma cell lines	[33]
ATRA	-	DOTAP/CH	Passive	In vivo in mice, IV	Lung cancer	[34]
Daunorubicin + Cytarabine	VYXEOS	DSPG/DSPC/CH	Passive	IV, in vivo, FDA approved	Secondary acute myeloid leukemia (sAML)	[15,35,36,37]
Paclitaxel	LEP-ETU	DOPC/CH/cardiolipin	Passive	IV, in vivo, FDA approved	Ovarian cancer	[38,39]
Vincristine	-	Sphingomyelin/CH	Passive	IV, in vivo, clinical	Philadelphia chromosome-negative (Ph-) acute lymphoblastic leukemia (ALL)	[40,41,42]
Verteporfin	Visudyne	DMPC/EPG	Passive	IV, in vivo, clinical	EGFR-mutated glioblastoma	[43,44,45]
DOX	ThermoDox	DPPC/MSPC/PEG 2000-DSPE	Passive	IV, in vivo, clinical	Hepatocellular carcinoma (HCC)	[46,47]
Paclitaxel	EndoTAG-1	DOTAP/DOPC	Passive	IV, in vivo, clinical	Pancreatic cancer	[38,48]
miR-34a	-	DOTAP/CH	Passive	IV, in vivo, clinical	Advanced solid tumors	[40,49,50,51]
Irinotecan	ONIVYDE	DSPC/DSPE/CH/mPEG-2000	Passive	IV, in vivo, FDA approved	Metastatic adenocarcinoma of the pancreas	[52,53]
Mitomycin-C prodrug	Promitil	HSPC/CH/DSPE-PEG	Passive	IV, in vivo, clinical	Solid tumors	[54,55,56]
TUSC2/FUS1	REQORSA	DOTAP/CH	Passive	IV, in vivo, clinical	Non-Small cell lung cancer	[57,58]
Eribulin mesylate	E7389-LF	HSPC/CH/PEG 2000-DSPE	Passive	IV, in vivo, clinical	Solid tumors	[15,59,60]
Navelbine	-	DSPC/CH/PEG -DSPE	Passive	In vivo in mice	Colorectal cancer cells	[61]
Curcumin	Lipocurc	DMPG/DMPC	Passive	IV, in vivo, clinical	Metastatic tumors	[62,63,64]
Paclitaxel	PTX–LDE	Cholesteryl oleate/Egg-PC/Miglyol 812/CH	Passive	IV, in vivo, clinical	Ovarian carcinoma	[65,66,67]
PKN3 siRNA	Atu027	AtuFECT01/DPhyPE/DSPE-PEG-2000	Passive	IV, in vivo, clinical	Pancreatic cancer	[25]

Abbreviation: Hwtp53, human wild type p53; DNA, Deoxyribonucleic acid; DOTAP, 1,2-Dioleoyl-3-trimethylammonium-propane; DOPE, Dioleoyl phosphatidylethanolamine; scFv, single-chain variable fragment; IV, Intravenous; CH, Cholesterol; DOX, Doxorubicin; DSPC, Distearoyl phosphatidylcholine; DSPE, Distearoyl phosphoethanolamine; mPEG, methoxy Polyethylene Glycol; EGFR, Epidermal growth factor receptor; mAb, Monoclonal antibody; HSPC, Hydrogenated soybean phosphatidylcholine; PEG, Polyethylene Glycol; HER 2, Human epidermal growth factor receptor 2; POPC, palmitoyloleoyl phosphocholine; Ni-3NTA-DTDA, nitrilotriacetic acid ditetradecylamine, nickel salt; dAb, Single domain antibody; VH, variable heavy chain; DOPC, Dioleoyl phosphocholine; ATRA, all-trans-retinoic acid; DPPC, Dipalmitoyl phosphatidylcholine; DSPG, Distearoyl phosphoglycerol; DMPC, Dimyristoyl phosphocholine; EPC, egg phosphatidylglycerol; MSPC, Myristoyl-palmitoyl phosphatidylcholine; DMPG, Dimyristoyl phosphorylglycerol; Egg-PC, Egg phosphatidylcholine; PKN3, Protein Kinase N3; AtuFECT01, β-L-arginyl-2,3-L-diaminopropionic acid-N-palmityl-N-oleyl-amide trihydrochloride; DPhyPE, Diphytanoyl phosphoethanolamine.

**Table 2:** Challenges common to both active and passive targeting.

	Active and Passive Targeting Challenges
1.	Scale up liposome preparation to reproducibly achieve target product profile including in vitro drug release rate, particle size distribution, lamellarity, stability, drug encapsulation efficiency, etc.
2.	Separation of raw lipids in a mixture of lipids and ability to analyze them
3.	Determination of complete stability and toxicity profile of novel lipids involved in formulations
4.	Stability of liposomes in solution
5.	Determination of biodistribution of liposomes appropriate PK/PD models to predict parameters in humans
6.	Immunogenic reactions such as CARPA upon IV administration of liposomes have resulted in additional layer of challenge

Abbreviations: PK, Pharmacokinetics; PD, Pharmacodynamics; CARPA, Complement activation-related pseudoallergy; IV, Intravenous.

## Data Availability

Data sharing not applicable.

## References

[1] https://web.archive.org/web/20210707200955/https://gicr.iarc.fr/public/docs/20120906-WorldCancerFactSheet.pdf.

[2] https://web.archive.org/web/20210707201547/https://gco.iarc.fr/today/data/factsheets/cancers/39-All-cancers-fact-sheet.pdf.

[3] https://web.archive.org/web/20210707201915/https://www.cancer.org/content/dam/cancer-org/research/cancer-facts-and-statistics/annual-cancer-facts-and-figures/2021/cancer-facts-and-figures-2021.pdf.

[4] Gyanani V. (2013). Turning Stealth Liposomes into Cationic Liposomes for Anticancer Drug Delivery. Ph.D. Thesis.

[5] Allen T.M. (2002). Ligand-targeted therapeutics in anticancer therapy. Nat. Rev. Cancer.

[6] Yao X., Panichpisal K., Kurtzman N., Nugent K. (2007). Cisplatin nephrotoxicity: A review. Am. J. Med. Sci..

[7] Pfeffer B., Tziros C., Katz R.J. (2009). Current concepts of anthracycline cardiotoxicity: Pathogenesis, diagnosis and prevention. Br. J. Cardiol..

[8] Partridge A.H., Burstein H.J., Winer E.P. (2001). Side effects of chemotherapy and combined chemohormonal therapy in women with early-stage breast cancer. J. Natl. Cancer Inst. Monogr..

[9] Fabbrocini G., Cameli N., Romano M.C., Mariano M., Panariello L., Bianca D., Giuseppe M. (2012). Chemotherapy and skin reactions. J. Exp. Clin. Cancer Res..

[10] Gottesman M.M., Fojo T., Bates S.E. (2002). Multidrug resistance in cancer: Role of ATP–dependent transporters. Nat. Rev. Cancer.

[11] Bentzen S.M. (2006). Preventing or reducing late side effects of radiation therapy: Radiobiology meets molecular pathology. Nat. Rev. Cancer.

[12] Bosslet K., Straub R., Blumrich M., Czech J., Gerken M., Sperker B., Kroemer H.K., Gesson J.P., Koch M., Monneret C. (1998). Elucidation of the mechanism enabling tumor selective prodrug monotherapy. Cancer Res..

[13] Senzer N., Nemunaitis J., Nemunaitis D., Bedell C., Edelman G., Barve M., Nunan R., Pirollo K.F., Rait A., Chang E.H. (2012). Results of a Phase I Trial of SGT-53: A Systemically Administered, Tumor-Targeting Immunoliposome Nanocomplex Incorporating a Plasmid Encoding wtp53. Clin. Gene Cell Ther. Oral Abstr. Sess..

[14] Pirollo K.F., Nemunaitis J., Leung P.K., Nunan R., Adams J., Chang E.H. (2016). Safety and Efficacy in Advanced Solid Tumors of a Targeted Nanocomplex Carrying the p53 Gene Used in Combination with Docetaxel: A Phase 1b Study. Mol. Ther..

[15] Kim E.M., Jeong H.J. (2021). Liposomes: Biomedical Applications. Chonnam. Med. J..

[16] https://clinicaltrials.gov/ct2/show/NCT03076372.

[17] Moles E., Kavallaris M. (2019). A potent targeted cancer nanotherapeutic. Nat. Biomed. Eng..

[18] Wang D., Sun Y., Liu Y., Meng F., Lee R.J. (2018). Clinical translation of immunoliposomes for cancer therapy: Recent perspectives. Expert Opin. Drug Deliv..

[19] Yan W., Leung S.S., To K.K. (2020). Updates on the use of liposomes for active tumor targeting in cancer therapy. Nanomedicine.

[20] https://clinicaltrials.gov/ct2/show/NCT03603379.

[21] Paranthaman S., Goravinahalli Shivananjegowda M., Mahadev M., Moin A., Hagalavadi Nanjappa S., Nanjaiyah N., Chidambaram S.B., Gowda D.V. (2020). Nanodelivery Systems Targeting Epidermal Growth Factor Receptors for Glioma Management. Pharmaceutics.

[22] Miller K., Cortes J., Hurvitz S.A., Krop I.E., Tripathy D., Verma S., Riahi K., Reynolds J.G., Wickham T.J., Molnar I. (2016). HERMIONE: A randomized Phase 2 trial of MM-302 plus trastuzumab versus chemotherapy of physician’s choice plus trastuzumab in patients with previously treated, anthracycline-naïve, HER2-positive, locally advanced/metastatic breast cancer. BMC Cancer.

[23] https://www.clinicaltrials.gov/ct2/show/NCT01304797.

[24] Gargett T., Abbas M.N., Rolan P., Price J.D., Gosling K.M., Ferrante A., Ruszkiewicz A., Atmosukarto I.I.C., Altin J., Parish C.R. (2018). Phase I trial of Lipovaxin-MM, a novel dendritic cell-targeted liposomal vaccine for malignant melanoma. Cancer Immunol. Immunother..

[25] Liu C., Zhang L., Zhu W., Guo R., Sun H., Chen X., Deng N. (2020). Barriers and Strategies of Cationic Liposomes for Cancer Gene Therapy. Mol. Ther.-Methods Clin. Dev..

[26] https://www.clinicaltrials.gov/ct2/show/NCT01517464.

[27] Siefker-Radtke A., Zhang X.Q., Guo C.C., Shen Y., Pirollo K.F., Sabir S., Leung C., Leong-Wu C., Ling C.M., Chang E.H. (2016). A Phase l Study of a Tumor-targeted Systemic Nanodelivery System, SGT-94, in Genitourinary Cancers. Mol. Ther..

[28] https://www.cancer.gov/publications/dictionaries/cancer-drug/def/748730.

[29] https://patents.justia.com/patent/20180334724.

[30] Song X.L., Liu S., Jiang Y., Gu L.Y., Xiao Y., Wang X., Cheng L., Li X.T. (2017). Targeting vincristine plus tetrandrine liposomes modified with DSPE-PEG(2000)-transferrin in treatment of brain glioma. Eur. J. Pharm. Sci..

[31] Chiani M., Norouzian D., Shokrgozar M.A., Azadmanesh K., Najmafshar A., Mehrabi M.R., Akbarzadeh A. (2018). Folic acid conjugated nanoliposomes as promising carriers for targeted delivery of bleomycin. Artif. Cells Nanomed. Biotechnol..

[32] Della Giovampaola C., Capone A., Ermini L., Lupetti P., Vannuccini E., Finetti F., Donnini S., Ziche M., Magnani A., Leone G. (2017). Formulation of liposomes functionalized with Lotus lectin and effective in targeting highly proliferative cells. Biochim Biophys. Acta Gen. Subj..

[33] Cristiano M.C., Cosco D., Celia C., Tudose A., Mare R., Paolino D., Fresta M. (2017). Anticancer activity of all-trans retinoic acid-loaded liposomes on human thyroid carcinoma cells. Colloids Surf. B Biointerfaces.

[34] Grace V.M., Viswanathan S. (2017). Pharmacokinetics and therapeutic efficiency of a novel cationic liposome nano-formulated all trans retinoic acid in lung cancer mice model. J. Drug Deliv. Sci. Technol..

[35] Lancet J.E., Uy G.L., Cortes J.E., Newell L.F., Lin T.L., Ritchie E.K., Stuart R.K., Strickland S.A., Hogge D., Solomon S.R. (2018). CPX-351 (cytarabine and daunorubicin) Liposome for Injection Versus Conventional Cytarabine Plus Daunorubicin in Older Patients with Newly Diagnosed Secondary Acute Myeloid Leukemia. J. Clin. Oncol..

[36] Pelzer U., Blanc J.F., Melisi D., Cubillo A., Von Hoff D.D., Wang-Gillam A., Chen L.T., Siveke J.T., Wan Y., Solem C.T. (2017). Quality-adjusted survival with combination nal-IRI+5-FU/LV vs. 5-FU/LV alone in metastatic pancreatic cancer patients previously treated with gemcitabine-based therapy: A Q-TWiST analysis. Br. J. Cancer.

[37] Tran S., DeGiovanni P.J., Piel B., Rai P. (2017). Cancer nanomedicine: A review of recent success in drug delivery. Clin. Transl. Med..

[38] Bulbake U., Doppalapudi S., Kommineni N., Khan W. (2017). Liposomal Formulations in Clinical Use: An Updated Review. Pharmaceutics.

[39] Lamichhane N., Udayakumar T.S., D’Souza W.D., Simone C.B., Raghavan S.R., Polf J., Mahmood J. (2018). Liposomes: Clinical Applications and Potential for Image-Guided Drug Delivery. Molecules.

[40] Bozzuto G., Molinari A. (2015). Liposomes as nanomedical devices. Int. J. Nanomed..

[41] http://web.archive.org/web/20210813002334/https://patents.google.com/patent/AU2013347990A1/en.

[42] http://web.archive.org/web/20210813001653/http://www.druginformation.com/rxdrugs/V/VinCRIStine%20Sulfate%20LIPOSOME%20Injection.html.

[43] http://web.archive.org/web/20210813003036/https://www.bausch.com/ecp/our-products/rx-pharmaceuticals/rx-pharmaceuticals/visudyne-verteporfin-for-injection.

[44] Ghosh S., Carter K.A., Lovell J.F. (2019). Liposomal formulations of photosensitizers. Biomaterials.

[45] https://web.archive.org/web/20210422023716/https://clinicaltrials.gov/ct2/show/NCT04590664.

[46] https://web.archive.org/web/20210813005341/https://clinicaltrials.gov/ct2/show/NCT00617981.

[47] Lombardo D., Calandra P., Barreca D., Magazù S., Kiselev M.A. (2016). Soft Interaction in Liposome Nanocarriers for Therapeutic Drug Delivery. Nanomaterials.

[48] http://web.archive.org/web/20210813010747/https://www.syncorebio.com/en/sb05pc-endotag-1-phase-iii-been-approved-in-china-by-nmpa/.

[49] Hong D.S., Kang Y.-K., Borad M., Sachdev J., Ejadi S., Lim H.Y., Brenner A.J., Park K., Lee J.L., Kim T.Y. (2020). Phase 1 study of MRX34, a liposomal miR-34a mimic, in patients with advanced solid tumors. Br. J. Cancer.

[50] Lin X., Chen W., Wei F., Zhou B.P., Hung M.C., Xie X. (2017). Nanoparticle Delivery of miR-34a Eradicates Long-term-cultured Breast Cancer Stem Cells via Targeting C22ORF28 Directly. Theranostics.

[51] Shi J., Kantoff P.W., Wooster R., Farokhzad O.C. (2017). Cancer nanomedicine: Progress, challenges and opportunities. Nat. Rev. Cancer.

[52] https://web.archive.org/web/20210813021139/https://www.onivyde.com/websites/onivyde_us_online/wp-content/uploads/sites/3/2018/12/14105740/ONIVYDE_USPI.pdf.

[53] http://web.archive.org/web/20210225052514if_/https://www.curetoday.com/view/onivyde-shows-promise-in-patients-with-small-cell-lung-cancer-who-become-resistant-to-chemotherapy.

[54] Gabizon A., Shmeeda H., Tahover E., Kornev G., Patil Y., Amitay Y., Ohana P., Sapir E., Zalipsky S. (2020). Development of Promitil^®^, a lipidic prodrug of mitomycin c in PEGylated liposomes: From bench to bedside. Adv. Drug Deliv. Rev..

[55] http://lipomedix.com/Products/%C2%AEPromitil.

[56] http://web.archive.org/web/20210813024839/https://www.globenewswire.com/news-release/2020/01/23/1974347/0/en/LipoMedix-Announces-Publication-of-Positive-Phase-1-Data-for-Promitil-PL-MLP-in-Research-Journal-Investigational-New-Drugs.html.

[57] https://web.archive.org/web/20210813025653/https://www.genprex.com/technology/reqorsa/.

[58] https://web.archive.org/web/20210813030047/https://adisinsight.springer.com/drugs/800018766.

[59] https://web.archive.org/web/20210813030656/https://clinicaltrials.gov/ct2/show/NCT04078295.

[60] https://web.archive.org/web/20210813031053/https://ascopubs.or.

[61] Chien Y.C., Chou Y.H., Wang W.H., Chen J.C., Chang W.S., Tsai C.W., Bau D.T., Hwang J.J. (2020). Therapeutic Efficacy Evaluation of Pegylated Liposome Encapsulated with Vinorelbine Plus (111) in Repeated Treatments in Human Colorectal Carcinoma with Multimodalities of Molecular Imaging. Cancer Genom. Proteom..

[62] Greil R., Greil-Ressler S., Weiss L., Schönlieb C., Magnes T., Radl B., Bolger G.T., Vcelar B., Sordillo P.P. (2018). A phase 1 dose-escalation study on the safety, tolerability and activity of liposomal curcumin (Lipocurc(™)) in patients with locally advanced or metastatic cancer. Cancer Chemother. Pharmacol..

[63] Bolger G.T., Licollari A., Tan A., Greil R., Vcelar B., Majeed M., Helson L. (2017). Distribution and Metabolism of Lipocurc™ (Liposomal Curcumin) in Dog and Human Blood Cells: Species Selectivity and Pharmacokinetic Relevance. Anticancer Res..

[64] https://web.archive.org/web/20210813032817/https://apnews.com/press-release/pr-businesswire/eb9457dc9500491e918b53fdeeb75494.

[65] Graziani S.R., Vital C.G., Morikawa A.T., Van Eyll B.M., Fernandes Junior H.J., Kalil Filho R., Maranhão R.C. (2017). Phase II study of paclitaxel associated with lipid core nanoparticles (LDE) as third-line treatment of patients with epithelial ovarian carcinoma. Med. Oncol..

[66] Occhiutto M.L., Freitas F.R., Lima P.P., Maranhão R.C., Costa V.P. (2016). Paclitaxel Associated with Lipid Nanoparticles as a New Antiscarring Agent in Experimental Glaucoma Surgery. Investig. Ophthalmol. Vis. Sci..

[67] Gomes F.L.T., Maranhão R.C., Tavares E.R., Carvalho P.O., Higuchi M.L., Mattos F.R., Pitta F.G., Hatab S.A., Kalil-Filho R., Serrano C.V. (2018). Regression of Atherosclerotic Plaques of Cholesterol-Fed Rabbits by Combined Chemotherapy with Paclitaxel and Methotrexate Carried in Lipid Core Nanoparticles. J. Cardiovasc. Pharmacol. Ther..

[68] Peer D., Karp J.M., Hong S., Farokhzad O.C., Margalit R., Langer R. (2007). Nanocarriers as an emerging platform for cancer therapy. Nat. Nanotechnol..

[69] Adams G.P., Schier R., McCall A.M., Simmons H.H., Horak E.M., Alpaugh R.K., Marks J.D., Weiner L.M. (2001). High affinity restricts the localization and tumor penetration of single-chain fv antibody molecules. Cancer Res..

[70] Tolcher A.W., Sugarman S., Gelmon K.A., Cohen R., Saleh M., Isaacs C., Young L., Healey D., Onetto N., Slichenmyer W. (1999). Randomized phase II study of BR96-doxorubicin conjugate in patients with metastatic breast cancer. J. Clin. Oncol..

[71] Kamoun W.S., Kirpotin D.B., Huang Z.R., Tipparaju S.K., Noble C.O., Hayes M.E., Luus L., Koshkaryev A., Kim J., Olivier K. (2019). Antitumour activity and tolerability of an EphA2-targeted nanotherapeutic in multiple mouse models. Nat. Biomed. Eng..

[72] Matusewicz L., Filip-Psurska B., Psurski M., Tabaczar S., Podkalicka J., Wietrzyk J., Ziółkowski P., Czogalla A., Sikorski A.F. (2019). EGFR-targeted immunoliposomes as a selective delivery system of simvastatin, with potential use in treatment of triple-negative breast cancers. Int. J. Pharm..

[73] Khayrani A.C., Mahmud H., Oo A.K.K., Zahra M.H., Oze M., Du J., Alam M.J., Afify S.M., Quora H.A.A., Shigehiro T. (2019). Targeting Ovarian Cancer Cells Overexpressing CD44 with Immunoliposomes Encapsulating Glycosylated Paclitaxel. Int. J. Mol. Sci..

[74] Hua S. (2019). Synthesis and in vitro characterization of oxytocin receptor targeted PEGylated immunoliposomes for drug delivery to the uterus. J. Liposome Res..

[75] Takahara M., Kamiya N. (2020). Synthetic Strategies for Artificial Lipidation of Functional Proteins. Chem.-A Eur. J..

[76] Marcos-Contreras O.A., Greineder C.F., Kiseleva R.Y., Parhiz H., Walsh L.R., Zuluaga-Ramirez V., Myerson J.W., Hood E.D., Villa C.H., Tombacz I. (2020). Selective targeting of nanomedicine to inflamed cerebral vasculature to enhance the blood–brain barrier. Proc. Natl. Acad. Sci. USA.

[77] Orleth A., Mamot C., Rochlitz C., Ritschard R., Alitalo K., Christofori G., Wicki A. (2016). Simultaneous targeting of VEGF-receptors 2 and 3 with immunoliposomes enhances therapeutic efficacy. J. Drug Target..

[78] Loureiro J.A., Gomes B., Fricker G., Cardoso I., Ribeiro C.A., Gaiteiro C., Coelho M.A., Pereira Mdo C., Rocha S. (2015). Dual ligand immunoliposomes for drug delivery to the brain. Colloids Surf. B Biointerfaces.

[79] Paul J.W., Hua S., Ilicic M., Tolosa J.M., Butler T., Robertson S., Smith R. (2017). Drug delivery to the human and mouse uterus using immunoliposomes targeted to the oxytocin receptor. Am. J. Obstet. Gynecol..

[80] Moles E., Urbán P., Jiménez-Díaz M.B., Viera-Morilla S., Angulo-Barturen I., Busquets M.A., Fernàndez-Busquets X. (2015). Immunoliposome-mediated drug delivery to Plasmodium-infected and non-infected red blood cells as a dual therapeutic/prophylactic antimalarial strategy. J. Control. Release.

[81] Moles E., Galiano S., Gomes A., Quiliano M., Teixeira C., Aldana I., Gomes P., Fernàndez-Busquets X. (2017). ImmunoPEGliposomes for the targeted delivery of novel lipophilic drugs to red blood cells in a falciparum malaria murine model. Biomaterials.

[82] Ramana L.N., Sharma S., Sethuraman S., Ranga U., Krishnan U.M. (2015). Stealth anti-CD4 conjugated immunoliposomes with dual antiretroviral drugs--modern Trojan horses to combat HIV. Eur. J. Pharm. Biopharm..

[83] Ding T., Guan J., Wang M., Long Q., Liu X., Qian J., Wei X., Lu W., Zhan C. (2020). Natural IgM dominates in vivo performance of liposomes. J. Control. Release.

[84] Rabenhold M., Steiniger F., Fahr A., Kontermann R.E., Rüger R. (2015). Bispecific single-chain diabody-immunoliposomes targeting endoglin (CD105) and fibroblast activation protein (FAP) simultaneously. J. Control. Release.

[85] Peng J., Chen J., Xie F., Bao W., Xu H., Wang H., Xu Y., Du Z. (2019). Herceptin-conjugated paclitaxel loaded PCL-PEG worm-like nanocrystal micelles for the combinatorial treatment of HER2-positive breast cancer. Biomaterials.

[86] Kraft J.C., Freeling J.P., Wang Z., Ho R.J.Y. (2014). Emerging research and clinical development trends of liposome and lipid nanoparticle drug delivery systems. J. Pharm. Sci..

[87] https://web.archive.org/web/20210728202637/https://www.sciencenews.org/article/coronavirus-covid-deadly-black-fungus-infection-india.

[88] Immordino M.L., Dosio F., Cattel L. (2006). Stealth liposomes: Review of the basic science, rationale, and clinical applications, existing and potential. Int. J. Nanomed..

[89] Scherphof G.L., Dijkstra J., Spanjer H.H., Derksen J.T., Roerdink F.H. (1985). Uptake and intracellular processing of targeted and nontargeted liposomes by rat Kupffer cells in vivo and in vitro. Ann. N. Y. Acad. Sci..

[90] Cullis P.R., Chonn A., Semple S.C. (1998). Interactions of liposomes and lipid-based carrier systems with blood proteins: Relation to clearance behaviour in vivo. Adv. Drug Deliv. Rev..

[91] Senior J.H., Trimble K.R., Maskiewicz R. (1991). Interaction of positively-charged liposomes with blood: Implications for their application in vivo. Biochim. Biophys. Acta.

[92] Oku N., Tokudome Y., Namba Y., Saito N., Endo M., Hasegawa Y., Kawai M., Tsukada H., Okada S. (1996). Effect of serum protein binding on real-time trafficking of liposomes with different charges analyzed by positron emission tomography. Biochim. Biophys. Acta.

[93] Philippot J.R., Schube F. (1994). Liposomes as Tools in Basic Research and Industry.

[94] Du H., Chandaroy P., Hui S.W. (1997). Grafted poly-(ethylene glycol) on lipid surfaces inhibits protein adsorption and cell adhesion. Biochim. Biophys. Acta.

[95] Hong R.L., Huang C.J., Tseng Y.L., Pang V.F., Chen S.T., Liu J.J., Chang F.H. (1999). Direct comparison of liposomal doxorubicin with or without polyethylene glycol coating in C-26 tumor-bearing mice: Is surface coating with polyethylene glycol beneficial?. Clin. Cancer Res..

[96] Parr M.J., Masin D., Cullis P.R., Bally M.B. (1997). Accumulation of liposomal lipid and encapsulated doxorubicin in murine Lewis lung carcinoma: The lack of beneficial effects by coating liposomes with poly(ethylene-glycol). J. Pharmacol. Exp. Ther..

[97] Kale A.A., Torchilin V.P. (2007). Design, Synthesis, and Characterization of pH-Sensitive PEG−PE Conjugates for Stimuli-Sensitive Pharmaceutical Nanocarriers:  The Effect of Substitutes at the Hydrazone Linkage on the pH Stability of PEG−PE Conjugates. Bioconjugate Chem..

[98] Sun Y., Zhao D., Wang G., Wang Y., Cao L., Sun J., Jiang Q., He Z. (2020). Recent progress of hypoxia-modulated multifunctional nanomedicines to enhance photodynamic therapy: Opportunities, challenges, and future development. Acta Pharm. Sin. B.

[99] Sharma A., Arambula J.F., Koo S., Kumar R., Singh H., Sessler J.L., Kim J.S. (2019). Hypoxia-targeted drug delivery. Chem. Soc. Rev..

[100] Andresen T.L., Thompson D.H., Kaasgaard T. (2010). Enzyme-triggered nanomedicine: Drug release strategies in cancer therapy (Invited Review). Mol. Membr. Biol..

[101] Li S., Zou Q., Xing R., Govindaraju T., Fakhrullin R., Yan X. (2019). Peptide-modulated self-assembly as a versatile strategy for tumor supramolecular nanotheranostics. Theranostics.

[102] Horsman M.R., Vaupel P. (2016). Pathophysiological Basis for the Formation of the Tumor Microenvironment. Front. Oncol..

[103] Kalafatovic D., Nobis M., Son J., Anderson K.I., Ulijn R.V. (2016). MMP-9 triggered self-assembly of doxorubicin nanofiber depots halts tumor growth. Biomaterials.

[104] Zhang N., Zhao F., Zou Q., Li Y., Ma G., Yan X. (2016). Multitriggered Tumor-Responsive Drug Delivery Vehicles Based on Protein and Polypeptide Coassembly for Enhanced Photodynamic Tumor Ablation. Small.

[105] Canavese G., Ancona A., Racca L., Canta M., Dumontel B., Barbaresco F., Limongi T., Cauda V. (2018). Nanoparticle-assisted ultrasound: A special focus on sonodynamic therapy against cancer. Chem. Eng. J..

[106] Sirsi S.R., Borden M.A. (2014). State-of-the-art materials for ultrasound-triggered drug delivery. Adv. Drug Deliv. Rev..

[107] VanOsdol J., Ektate K., Ramasamy S., Maples D., Collins W., Malayer J., Ranjan A. (2017). Sequential HIFU heating and nanobubble encapsulation provide efficient drug penetration from stealth and temperature sensitive liposomes in colon cancer. J. Control. Release.

[108] Huang S.L., MacDonald R.C. (2004). Acoustically active liposomes for drug encapsulation and ultrasound-triggered release. Biochim. Biophys. Acta.

[109] Rapoport N. (2004). Combined cancer therapy by micellar-encapsulated drug and ultrasound. Int. J. Pharm..

[110] Leung S.J., Romanowski M. (2012). Light-Activated Content Release from Liposomes. Theranostics.

[111] Pidgeon C., Hunt C.A. (1983). Light sensitive liposomes. Photochem. Photobiol..

[112] Ohya Y., Okuyama Y., Fukunaga A., Ouchi T. (1998). Photo-sensitive lipid membrane perturbation by a single chain lipid having terminal spiropyran group. Supramol. Chem..

[113] Anderson V.C., Thompson D.H. (1992). Triggered release of hydrophilic agents from plasmalogen liposomes using visible light or acid. Biochim. Biophys. Acta.

[114] Thompson D.H., Gerasimov O.V., Wheeler J.J., Rui Y., Anderson V.C. (1996). Triggerable plasmalogen liposomes: Improvement of system efficiency. Biochim. Biophys. Acta.

[115] Wan Y., Angleson J.K., Kutateladze A.G. (2002). Liposomes from Novel Photolabile Phospholipids:  Light-Induced Unloading of Small Molecules as Monitored by PFG NMR. J. Am. Chem. Soc..

[116] Li Z., Wan Y., Kutateladze A.G. (2003). Dithiane-based photolabile amphiphiles: Toward photolabile liposomes. Langmuir.

[117] Zhang Z., Smith B.D. (1999). Synthesis and Characterization of NVOC-DOPE, a Caged Photoactivatable Derivative of Dioleoylphosphatidylethanolamine. Bioconjugate Chem..

[118] Bayer A.M., Alam S., Mattern-Schain S.I., Best M.D. (2014). Triggered liposomal release through a synthetic phosphatidylcholine analogue bearing a photocleavable moiety embedded within the sn-2 acyl chain. Chemistry.

[119] O’Brien D.F., Armitage B., Benedicto A., Bennett D.E., Lamparski H.G., Lee Y., Srisiri W., Sisson T.M. (1998). Polymerization of Preformed Self-Organized Assemblies. Acc. Chem. Res..

[120] Yavlovich A., Singh A., Blumenthal R., Puri A. (2011). A novel class of photo-triggerable liposomes containing DPPC:DC_8,9_PC as vehicles for delivery of doxorubcin to cells. Biochim. Biophys. Acta Biomembr..

[121] Gerasimov O.V., Boomer J.A., Qualls M.M., Thompson D.H. (1999). Cytosolic drug delivery using pH- and light-sensitive liposomes. Adv. Drug Deliv. Rev..

[122] Fuse T., Tagami T., Tane M., Ozeki T. (2018). Effective light-triggered contents release from helper lipid-incorporated liposomes co-encapsulating gemcitabine and a water-soluble photosensitizer. Int. J. Pharm..

[123] Li Q., Li W., Di H., Luo L., Zhu C., Yang J., Yin X., Yin H., Gao J., Du Y. (2018). A photosensitive liposome with NIR light triggered doxorubicin release as a combined photodynamic-chemo therapy system. J. Control. Release.

[124] Yatvin M.B., Weinstein J.N., Dennis W.H., Blumenthal R. (1978). Design of liposomes for enhanced local release of drugs by hyperthermia. Science.

[125] Ta T., Porter T.M. (2013). Thermosensitive liposomes for localized delivery and triggered release of chemotherapy. J. Control. Release.

[126] Nibu Y., Inoue T., Motoda I. (1995). Effect of headgroup type on the miscibility of homologous phospholipids with different acyl chain lengths in hydrated bilayer. Biophys. Chem..

[127] Meshorer A., Prionas S.D., Fajardo L.F., Meyer J.L., Hahn G.M., Martinez A.A. (1983). The effects of hyperthermia on normal mesenchymal tissues. Application of a histologic grading system. Arch. Pathol. Lab. Med..

[128] Anyarambhatla G.R., Needham D. (1999). Enhancement of the Phase Transition Permeability of DPPC Liposomes by Incorporation of MPPC: A New Temperature-Sensitive Liposome for use with Mild Hyperthermia. J. Liposome Res..

[129] Banno B., Ickenstein L.M., Chiu G.N.C., Bally M.B., Thewalt J., Brief E., Wasan E.K. (2010). The functional roles of poly(ethylene glycol)-lipid and lysolipid in the drug retention and release from lysolipid-containing thermosensitive liposomes in vitro and in vivo. J. Pharm. Sci..

[130] Sandström M.C., Ickenstein L.M., Mayer L.D., Edwards K. (2005). Effects of lipid segregation and lysolipid dissociation on drug release from thermosensitive liposomes. J. Control. Release.

[131] https://web.archive.org/web/20210728223201/https://www.globenewswire.com/en/news-release/2020/04/15/2016425/0/en/Celsion-Reports-that-Sufficient-Events-Have-Been-Reached-for-the-Second-Interim-Analysis-of-the-Phase-III-OPTIMA-Study-of-ThermoDox-in-Primary-Liver-Cancer.html.

[132] Swenson C.E., Haemmerich D., Maul D.H., Knox B., Ehrhart N., Reed R.A. (2015). Increased Duration of Heating Boosts Local Drug Deposition during Radiofrequency Ablation in Combination with Thermally Sensitive Liposomes (ThermoDox) in a Porcine Model. PLoS ONE.

[133] Amstad E., Kohlbrecher J., Müller E., Schweizer T., Textor M., Reimhult E. (2011). Triggered Release from Liposomes through Magnetic Actuation of Iron Oxide Nanoparticle Containing Membranes. Nano Lett..

[134] Babincová M., Cicmanec P., Altanerová V., Altaner C., Babinec P. (2002). AC-magnetic field controlled drug release from magnetoliposomes: Design of a method for site-specific chemotherapy. Bioelectrochemistry.

[135] Nobuto H., Sugita T., Kubo T., Shimose S., Yasunaga Y., Murakami T., Ochi M. (2004). Evaluation of systemic chemotherapy with magnetic liposomal doxorubicin and a dipole external electromagnet. Int. J. Cancer.

[136] Kubo T., Sugita T., Shimose S., Nitta Y., Ikuta Y., Murakami T. (2001). Targeted systemic chemotherapy using magnetic liposomes with incorporated adriamycin for osteosarcoma in hamsters. Int. J. Oncol..

[137] Babincová M., Altanerová V., Lampert M., Altaner C., Machová E., Srámka M., Babinec P. (2000). Site-specific in vivo targeting of magnetoliposomes using externally applied magnetic field. Z. Naturforsch. C J. Biosci..

[138] Matsunaga T., Higashi Y., Tsujimura N. (1997). Drug delivery by magnetoliposomes containing bacterial magnetic particles. Cell Eng..

[139] Jain S., Mishra V., Singh P., Dubey P.K., Saraf D.K., Vyas S.P. (2003). RGD-anchored magnetic liposomes for monocytes/neutrophils-mediated brain targeting. Int. J. Pharm..

[140] Smith J.A., Costales A.B., Jaffari M., Urbauer D.L., Frumovitz M., Kutac C.K., Tran H., Coleman R.L. (2016). Is it equivalent? Evaluation of the clinical activity of single agent Lipodox^®^ compared to single agent Doxil^®^ in ovarian cancer treatment. J. Oncol. Pharm. Pract..

[141] Bekersky I., Fielding R.M., Dressler D.E., Lee J.W., Buell D.N., Walsh T.J. (2002). Pharmacokinetics, excretion, and mass balance of liposomal amphotericin B (AmBisome) and amphotericin B deoxycholate in humans. Antimicrob. Agents Chemother..

[142] Charrois G.J.R., Allen T.M. (2004). Drug release rate influences the pharmacokinetics, biodistribution, therapeutic activity, and toxicity of pegylated liposomal doxorubicin formulations in murine breast cancer. Biochim. Biophys. Acta.

[143] Boswell G.W., Buell D., Bekersky I. (1998). AmBisome (liposomal amphotericin B): A comparative review. J. Clin. Pharmacol..

[144] Walsh T.J., Yeldandi V., McEvoy M., Gonzalez C., Chanock S., Freifeld A., Seibel N.I., Whitcomb P.O., Jarosinski P., Boswell G. (1998). Safety, Tolerance, and Pharmacokinetics of a Small Unilamellar Liposomal Formulation of Amphotericin B (AmBisome) in Neutropenic Patients. Antimicrob. Agents Chemother..

[145] Paolo D.D., Ambrogio C., Pastorino F., Brignole C., Martinengo C., Carosio R., Loi M., Pagnan G., Emionite L., Cilli M. (2011). Selective Therapeutic Targeting of the Anaplastic Lymphoma Kinase with Liposomal siRNA Induces Apoptosis and Inhibits Angiogenesis in Neuroblastoma. Mol. Ther..

[146] Cui J., Li C., Guo W., Li Y., Wang C., Zhang L., Zhang L., Hao Y., Wang Y. (2007). Direct comparison of two pegylated liposomal doxorubicin formulations: Is AUC predictive for toxicity and efficacy?. J. Control. Release.

[147] Cheung B.C.L., Sun T.H.T., Leenhouts J.M., Cullis P.R. (1998). Loading of doxorubicin into liposomes by forming Mn^2+^-drug complexes. Biochim. Biophys. Acta Biomembr..

[148] Szebeni J., Storm G. (2015). Complement activation as a bioequivalence issue relevant to the development of generic liposomes and other nanoparticulate drugs. Biochem. Biphys. Res. Commun..

[149] Szebeni J., Fishbane S., Hedenus M., Howaldt S., Locatelli F., Patni S., Rampton D., Weiss G., Folkersen J. (2015). Hypersensitivity to intravenous iron: Classification, terminology, mechanisms and management. Br. J. Pharmacol..

[150] Szebeni J., Muggia F., Barenholz Y., Dobrovolskaia M.A., McNeil S.E. (2016). Case Study: Complement Activation Related Hypersensitivity Reactions to PEGylated Liposomal Doxorubicin—Experimental and Clinical Evidence, Mechanisms and Approaches to Inhibition. Handbook of Immunological Properties of Engineered Nanomaterials.

[151] Kelly C., Lawlor C., Burke C., Barlow J.W., Ramsey J.M., Jefferies C., Cryan S. (2015). High-throughput methods for screening liposome-macrophage cell interaction. J. Liposome Res..

[152] Wientjes M.G., Yeung B.Z., Lu Z., Wientjes M.G., Au J.L.S. (2014). Predicting diffusive transport of cationic liposomes in 3-dimensional tumor spheroids. J. Control. Release.

